# Impaired bisecting GlcNAc reprogrammed M1 polarization of macrophage

**DOI:** 10.1186/s12964-023-01432-6

**Published:** 2024-01-26

**Authors:** Xin He, Bowen Wang, Wenli Deng, Jinhua Cao, Zengqi Tan, Xiang Li, Feng Guan

**Affiliations:** 1 Key Laboratory of Resource Biology and Biotechnology Western China, Ministry of Education; Provincial Key Laboratory of Biotechnology, College of Life Sciences, Northwest University, No, 229, Taibai North Road, Xi’an, Shaanxi 710069 China; 2 Institute of Hematology, School of Medicine, Northwest University, Xi’an, 710069 China

**Keywords:** Bisecting GlcNAc, Glycosylation, Macrophage, Polarization, LGALS3BP

## Abstract

The functions of macrophages are governed by distinct polarization phenotypes, which can be categorized as either anti-tumor/M1 type or pro-tumor/M2 type. Glycosylation is known to play a crucial role in various cellular processes, but its influence on macrophage polarization is not well-studied. In this study, we observed a significant decrease in bisecting GlcNAc during M0-M1 polarization, and impaired bisecting GlcNAc was found to drive M0-M1 polarization. Using a glycoproteomics strategy, we identified Lgals3bp as a specific glycoprotein carrying bisecting GlcNAc. A high level of bisecting GlcNAc modification facilitated the degradation of Lgals3bp, while a low level of bisecting GlcNAc stabilized Lgals3bp. Elevated levels of Lgals3bp promoted M1 polarization through the activation of the NF-кB pathway. Conversely, the activated NF-кB pathway significantly repressed the transcription of MGAT3, leading to reduced levels of bisecting GlcNAc modification on Lgals3bp. Overall, our study highlights the impact of glycosylation on macrophage polarization and suggests the potential of engineered macrophages via glycosylated modification.

Video Abstract

**Supplementary Information:**

The online version contains supplementary material available at 10.1186/s12964-023-01432-6.

## Background

Macrophages, as immune cells, have emerged as promising candidates for immunotherapy in the field of oncology [1]. Their functions are regulated by distinct polarization phenotypes, which can be categorized as either anti-tumor/M1 type or pro-tumor/M2 type [2]. The M1 phenotype enhances the immune response against tumors by promoting the Th1 response [3], presenting antigens to cytotoxic T cells [4] and releasing cytotoxic molecules such as nitric oxide (NO) [5] and reactive oxygen species (ROS) [6]. On the other hand, the M2 phenotype is characterized by the secretion of multiple anti-inflammatory cytokines such as IL-10, IL-13, and CCL22 [7], and it plays a role in tissue repair [8] and facilitates angiogenesis [9]. Additionally, nonpolarized M0 macrophages display significant plasticity, allowing them to transition into either M1 or M2 phenotypes [2]. This plasticity offers an attractive pathway for immunotherapy, as reprogramming M0 macrophages into the M1 phenotype holds promise for therapeutic intervention.

Various molecules, including proteins [7], lipids [10] and nucleic acids [11], contribute to the polarization of macrophages. For instance, NF-кB acts as a classical proinflammatory signaling molecule induced by lipopolysaccharide (LPS) to promote M1 polarization [12], while STAT6 is well-known for driving M2 polarization by enhancing the expression of M2-associated genes [13]. The interaction between CSF1 and CSF1R on macrophages leads to the polarization of macrophages towards the M2 phenotype [14]. Conversely, CSF1R inhibitors have been shown to repolarize tumor-associated macrophages (TAMs) into tumoricidal M1-like macrophages [15].

Recent studies have revealed that the polarization state of M1/M2 macrophages is associated with distinct glycomic signatures [16–18]. For example, during the M2 polarization process, the expression of α2,6 sialic acid and specific sialyltransferase genes associated with it is upregulated [18]. Aberrant glycosylation also plays a significant role in various diseases, particularly cancer, and alterations in glycosylation patterns between tumor cells and normal cells have been linked to tumor progression, immune dysregulation, and cell signaling disorders [19]. Furthermore, specific immunotherapeutic targets being investigated for macrophage-based therapies have been found to be glycosylated [20]. Inhibition of sialylation levels in TAMs has been shown to effectively reverse M2 macrophage polarization [21]. Additionally, elevated levels of O-GlcNAcylation in TAMs have been associated with M2 polarization [22], while the knockout of O-GlcNAc transferase (OGT) promotes proinflammatory polarization in macrophages [23]. Moreover, in cancer, increased sialylation directs macrophages toward the M2 phenotype by interacting with Siglec receptors on macrophages [24].

Bisecting GlcNAc, a specific and important form of N-glycosylation, plays a role in inhibiting N-glycan elongation [25]. Aberrant bisecting GlcNAc is commonly observed in cancer cells and serves as a potential diagnostic biomarker for cancer [26]. It has been observed that M2 macrophages exhibit elevated expression of bisecting GlcNAc compared to M0/M1 macrophages [18, 27, 28]. In our previous studies, we demonstrated that increased levels of bisecting GlcNAc significantly inhibit breast cancer growth and metastasis [29, 30]. Additionally, bisecting GlcNAc has been found to disrupt the interaction between stromal cells and myeloid cells [31]. However, the precise functional significance of bisecting GlcNAc in macrophage polarization remains uncertain.

In this study, we made an intriguing observation of decreased levels of bisecting GlcNAc during the process of M0-M1 macrophage polarization. This finding suggests that a deficiency in bisecting GlcNAc may contribute to driving this polarization. To gain further insights, we utilized proteomics and glycoproteomics methods to profile differentially expressed glycoproteins. Subsequently, we investigated the underlying mechanisms by which proteins carrying bisecting GlcNAc modulate macrophage polarization.

## Methods

### Cell lines and cell culture

All cell lines used in this study were obtained from the Cell Bank at the Chinese Academy of Sciences (Shanghai, China). Mouse macrophage cells RAW264.7 and human embryonic kidney cells HEK 293 T were cultured in DMEM (Biological Industries; Kibbutz Beit Haemek, Israel), while human monocytic leukemia cells THP-1, mouse colon cancer cells CT26, and mouse breast cancer cells 4T1 were cultured in RPMI 1640 (Biological Industries). All medium was supplemented with 10% fetal bovine serum (FBS) (Biological Industries).

The conditional medium (CM) was acquired by culturing RAW264.7 cells in a serum-free medium for 24 h. In the co-culture model, RAW264.7 cells were placed in 0.4 μm porous transwell inserts (Corning; Cambridge, MA, USA), while CT26 cells or 4T1 cells were seeded in the lower chamber.

### M0-M1 polarization

THP-1 cells were initially induced to differentiate into M0-type macrophages using 10 pmol/mL phorbol 12-myristate 13-acetate (PMA; Beyotime; Shanghai, China). Subsequently, these M0-type macrophages were stimulated to differentiate into M1-type macrophages by treatment with LPS (100 ng/mL) (Beyotime) and IFN-γ (20 ng/mL) (PeproTech; Rocky Hill, NJ, USA) for 24 h.

### Peritoneal macrophages

Peritoneal macrophages (PMs) were obtained following the previously described method [32]. Briefly, a total of 5 mL ice-cold PBS was injected into the peritoneal cavity of mice, followed by gentle abdominal massage for 5 min. The injected medium was subsequently collected and centrifuged at 1500 g for 8 min. The resulting cells were resuspended in complete medium and cultured for 24 h.

### Bone marrow-derived macrophages

Bone marrow mononuclear cells (BMMCs) were obtained following the previously described method [33]. Briefly, the mouse leg bone was penetrated and washed with RMPI 1640 medium. Cells were then cultured in red blood cell lysis buffer (TBD; Tianjin, China) for 2 min, and seeded onto 6-well plate. Adherent cells were treated with 20 ng/mL macrophages colony-stimulating factor (M-CSF; Beyotime) for 7 days to generate matured bone marrow-derived macrophages (BMDMs).

### Animal study

Balb/c mice aged 4–5 weeks were obtained from Cavens (Changzhou, China). A mixture of RAW264.7 cells and CT26 cells at a 1:1 ratio in 100 µL of PBS was subcutaneously injected into the interscapular region. After 4 weeks, the mice were euthanized, and tumor weight was recorded. Macrophages were isolated for further analysis using a previously described method [34].

### Glycoproteomics analysis

The glycopeptides were prepared following the protocol as described previously [35]. Briefly, cells were lysed using 8 M urea, 10 mM DTT, and 20 mM IAM (Sigma-Aldrich; St. Louis, MO, USA) with trypsin (Promega; Madison, WI, USA) for 4 h at 37 °C. Peptides were enriched and purified using Oasis HLB cartridges (Waters; Milford, MA, USA) and resuspended in 200 μL of 50% ACN with 1% TFA. The samples were then added to 4 mL of 95% ACN with 1% TFA, loaded and purified using Oasis MAX cartridges (Waters) to obtain glycopeptides. For immunoprecipitation-mass spectrometry (IP-MS), the protocol was performed as previously described [31]. Liquid chromatography coupled to tandem mass spectrometry (LC–MS/MS) was performed using an LTQ Orbitrap MS (Thermo Fisher Scientific; San Jose, CA, USA) and analyzed using the Byonic software program (Protein Metrics; San Carlos, CA, USA), Glyco-Decipher [36] and GlycoWorkbench 3.0.

### Data analysis

The GraphPad Prism V.7.0 (GraphPad Software; La Jolla, CA, USA) was used for statistical analysis. Data between two groups were compared using Student’s t test, and presented as the mean ± SEM. The differences at *p* < 0.05 were considered statistically significant. All experiment were performed in triplicate.

## Results

### Altered bisecting GlcNAc levels in M0-M1 polarization of macrophages

**Fig. 1:**
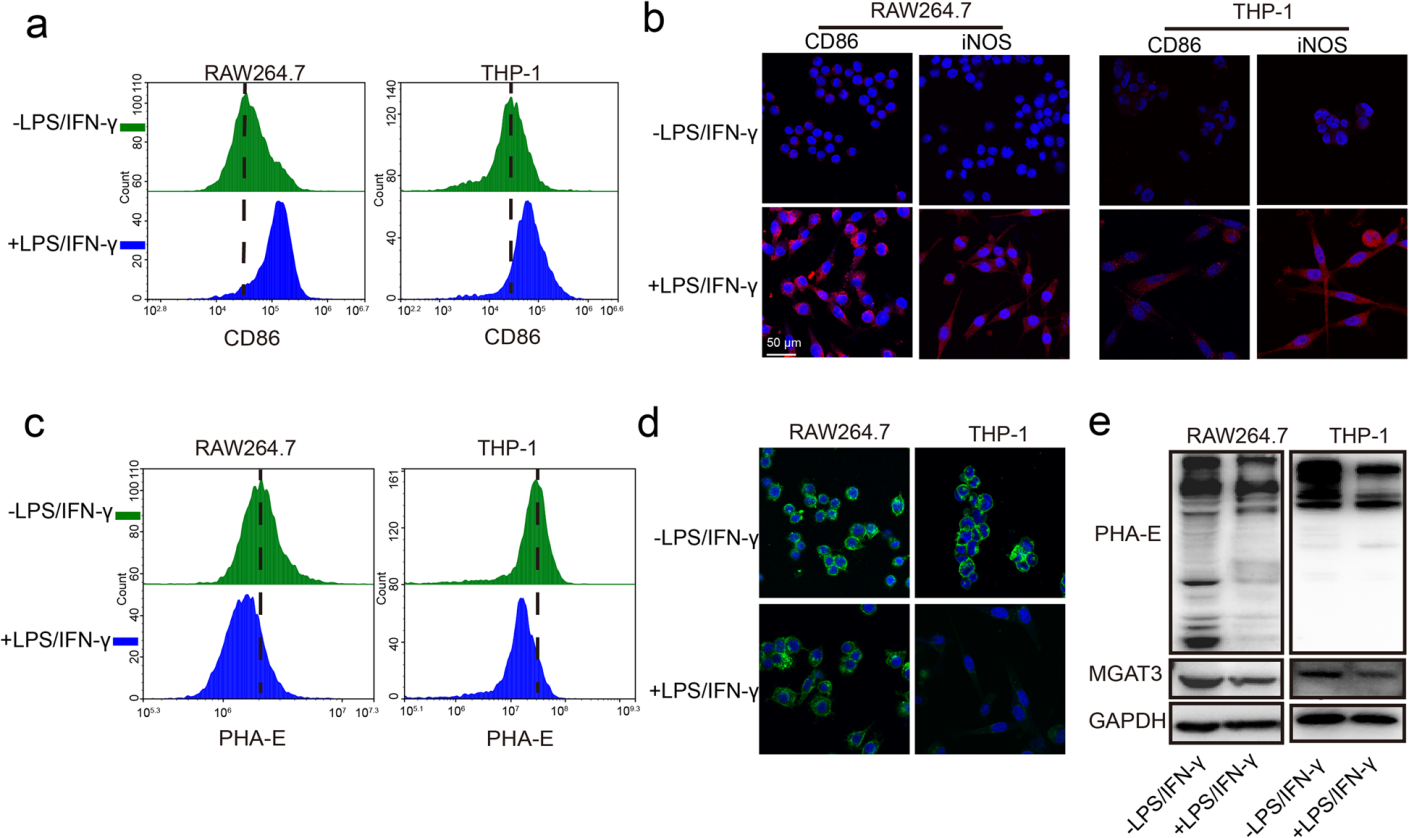
Bisecting GlcNAc levels in M0-M1 polarization. RAW264.7 and THP-1 cells were stimulated with LPS and IFN‐γ. **a** Expression of CD86 were checked by FACS. **b** Expression of CD86 and iNOS were checked by immunofluorescence. Bisecting GlcNAc levels were evaluated by FACS (**c**), immunofluorescence (**d**) and lectin blotting (**e**). MGAT3 expression was determined by western blotting (**e**)

Stimulation with LPS and IFN-γ caused polarization of the macrophage cells RAW264.7 and THP-1 from M0 type to M1 type. This was evidenced by enhanced proportion of CD86 positive cells (Fig. 1a), elevated expression of CD86 and iNOS (Fig. 1b), and increased the mRNA levels of CD86, TNF-α, iNOS, IL6 and IL1β (Fig. S1a).

Previous studies have reported that M2 phenotype exhibits higher levels of bisecting GlcNAc compared to M0/M1 phenotypes in macrophage cell lines, with lectin microarray [18] and glycoproteomics analysis [27]. Here, our observations revealed a significant reduction in bisecting GlcNAc levels in M1 macrophages compared to M0 macrophages, which was accompanied by a downregulation of MGAT3 expression (Fig. 1c-e). This decrease in bisecting GlcNAc levels was consistently observed in two primary macrophage cell types, mouse PMs and BMDMs, upon polarization to the M1 phenotype (Fig. S1b, c). This suggests that the reduction in bisecting GlcNAc levels is a common feature of M1 macrophages.

### Impaired bisecting GlcNAc modification drives the M1 polarization

**Fig. 2:**
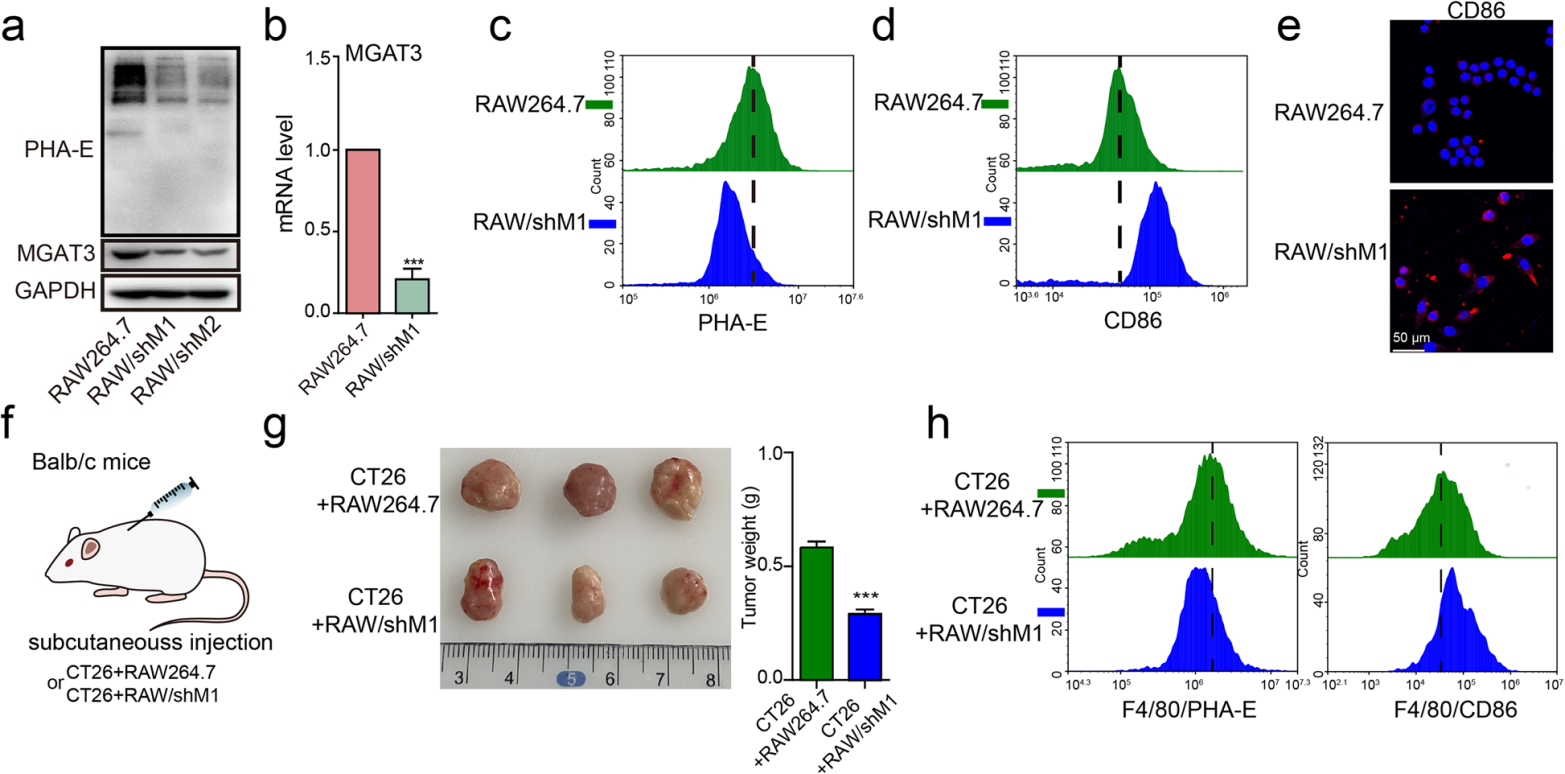
Effects of bisecting GlcNAc on M0-M1 polarization. MGAT3 was silenced in RAW264.7 cells (RAW/shM1/2). **a** Bisecting GlcNAc level and MGAT3 expression in RAW/shM1 cells by lectin/western blotting. **b** MGAT3 mRNA level in RAW/shM1 cells by qRT-PCR. **c**&**d** Bisecting GlcNAc level (**c**) and CD86 expression (**d**) in RAW/shM1 cells by FACS. **e** CD86 expression in RAW/shM1 cells by immunofluorescence. **f** Balb/c mice were subcutaneously injected with a mixture of CT26 cells and either RAW264.7 or RAW/shM1 cells. **g** Tumor weight. **h** Purified macrophages from tumor tissue stained by anti-F4/80, lectin PHA-E or anti-CD86

In order to further investigate the role of bisecting GlcNAc in macrophage polarization, we performed the silence of MGAT3 and observed a decrease in bisecting GlcNAc levels in corresponding RAW264.7 cells (RAW/shM1/2) (Fig. 2a-c, S2a). This reduction in bisecting GlcNAc levels was accompanied by an increase in CD86 expression (Fig. 2d, e) and an upregulation of mRNA levels for CD86, TNF-α, iNOS, IL6, and IL1β (Fig. S2b), suggesting that the reduction in bisecting GlcNAc levels in macrophages elicits effects similar to those induced by LPS and IFN-γ stimulation. Conversely, the introduction of MGAT3 into RAW264.7 cells (RAW/MGAT3) (Fig. S2c-f), hindered the M0-M1 polarization response upon treatment with LPS and IFN-γ (Fig. S2g, h).

**Fig. 3:**
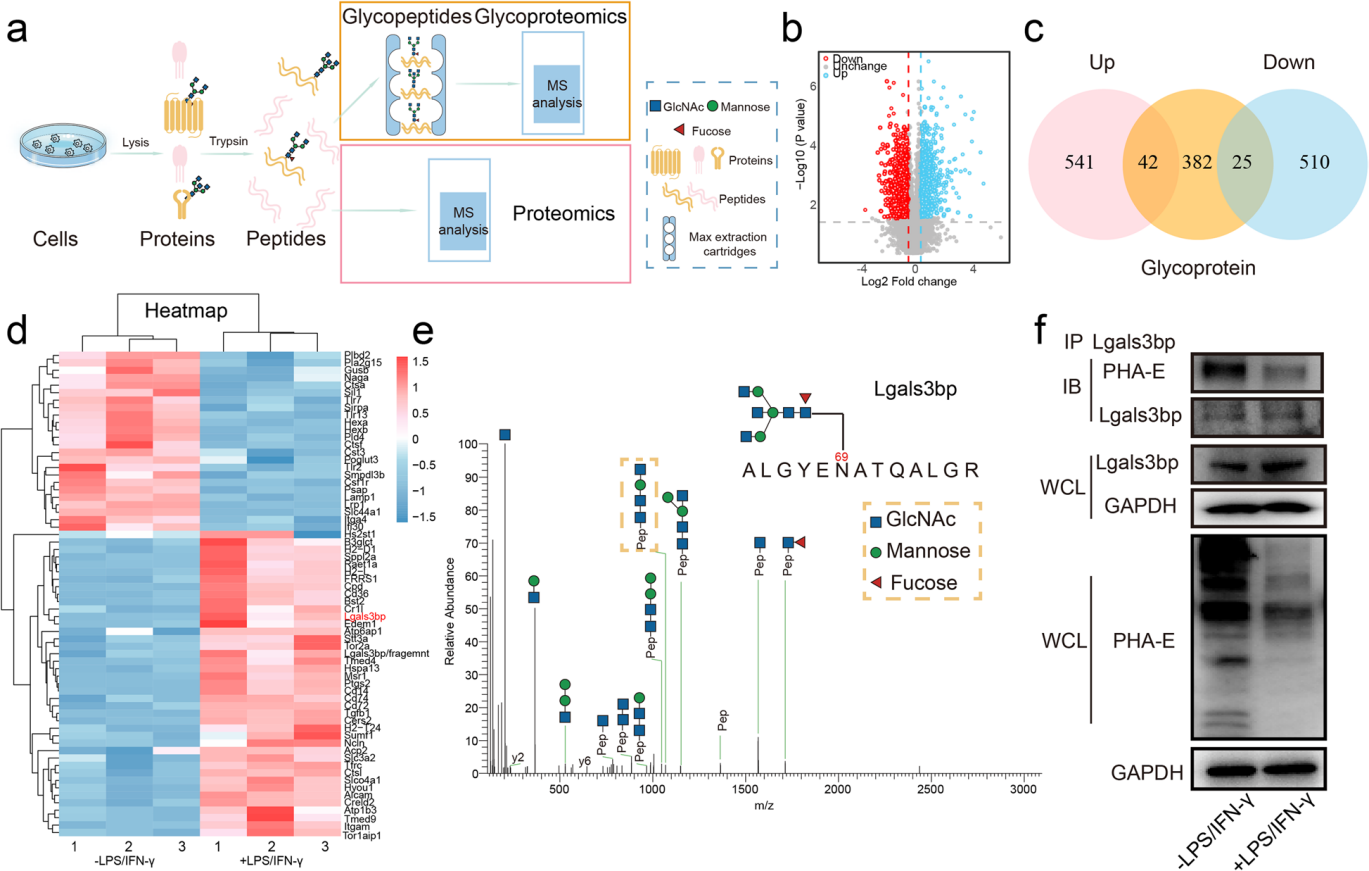
Lgals3bp identified as a bisecting GlcNAc modified glycoprotein. **a** Work flow of proteomics and glycoproteomics analysis by LC–MS/MS. **b** Volcano map of differentially expressed protein in M0-M1 polarization of RAW264.7 cells. **c, d** Differentially expressed glycoproteins in M0-M1 polarization of RAW264.7 cells by Venn diagram (**c**) and heatmap (**d**). **e** Representative MS/MS spectrum of ALGYEN#ATQALGR of Lgals3bp with bisecting GlcNAc in RAW264.7 cells. **f** Lgals3bp expression or its bisecting GlcNAc modification, and whole cellular level of bisecting GlcANc modification in M0-M1 polarization of RAW264.7 cells by western blotting or immunoprecipitation (IP). WCL, whole cell lysate

In the tumor microenvironment, M1 macrophages, which are immune-activated cells, have been shown to play a role in inhibiting tumor progression [37]. We found that RAW/shM1 cells demonstrated inhibitory effects on cell migration and proliferation (Fig. S3a-d), while simultaneously promoting apoptosis in 4T1 and CT26 cells (Fig. S3e). In a mouse model, the co-injection of RAW/shM1 cells resulted in a significant reduction in the tumor growth of CT26 cells (Fig. 2f&g, S3f). To investigate the changes in macrophage phenotype within the tumor microenvironment, we purified macrophages from the tumor tissue in the mixed RAW/shM1 group. These macrophages displayed a decreased proportion of bisecting GlcNAc and an elevated proportion of CD86-positive cells, indicating the M1-like polarization (Fig. 3h). Consistent with this, we also observed an upregulation of mRNA levels for CD86, TNF-α and iNOS (Fig. S3g).

### Lgals3bp bearing with bisecting GlcNAc in macrophages

Bisecting GlcNAc, as a classic N-glycan structure, is attached to certain glycoproteins. Consequently, using proteomics and glycoproteomics analysis, we investigated the proteins displaying differential expression and harboring the bisecting GlcNAc structure during the M0-M1 polarization of RAW264.7 cells (Fig. 3a). Out of the 1,118 differentially expressed proteins identified, 583 exhibited upregulation, whereas 535 displayed downregulation in M1 macrophages as compared to M0 macrophages (Fig. 3b). Within this dataset, we focused on glycoproteins and identified 42 upregulated glycoproteins and 25 downregulated glycoproteins (Fig. 3c, d).

To identify the presence of bisecting GlcNAc on glycopeptides, we focused on the distinctive ions [pep + N3H] and [pep + N3HF] [38], as observed in the galectin 3 binding protein (Lgals3bp) from glycoproteomics analysis. Using IP-MS, we confirmed that Lgals3bp has the distinctive ion [pep + N3H] at Asp 69 (Fig. 3e). Notably, the expression of Lgals3bp was increased in M1 type, which aligns with the observed decrease in bisecting GlcNAc levels on Lgals3bp and at whole cellular level (Fig. 3f).

### Effect of Lgals3bp on macrophage polarization

**Fig. 4:**
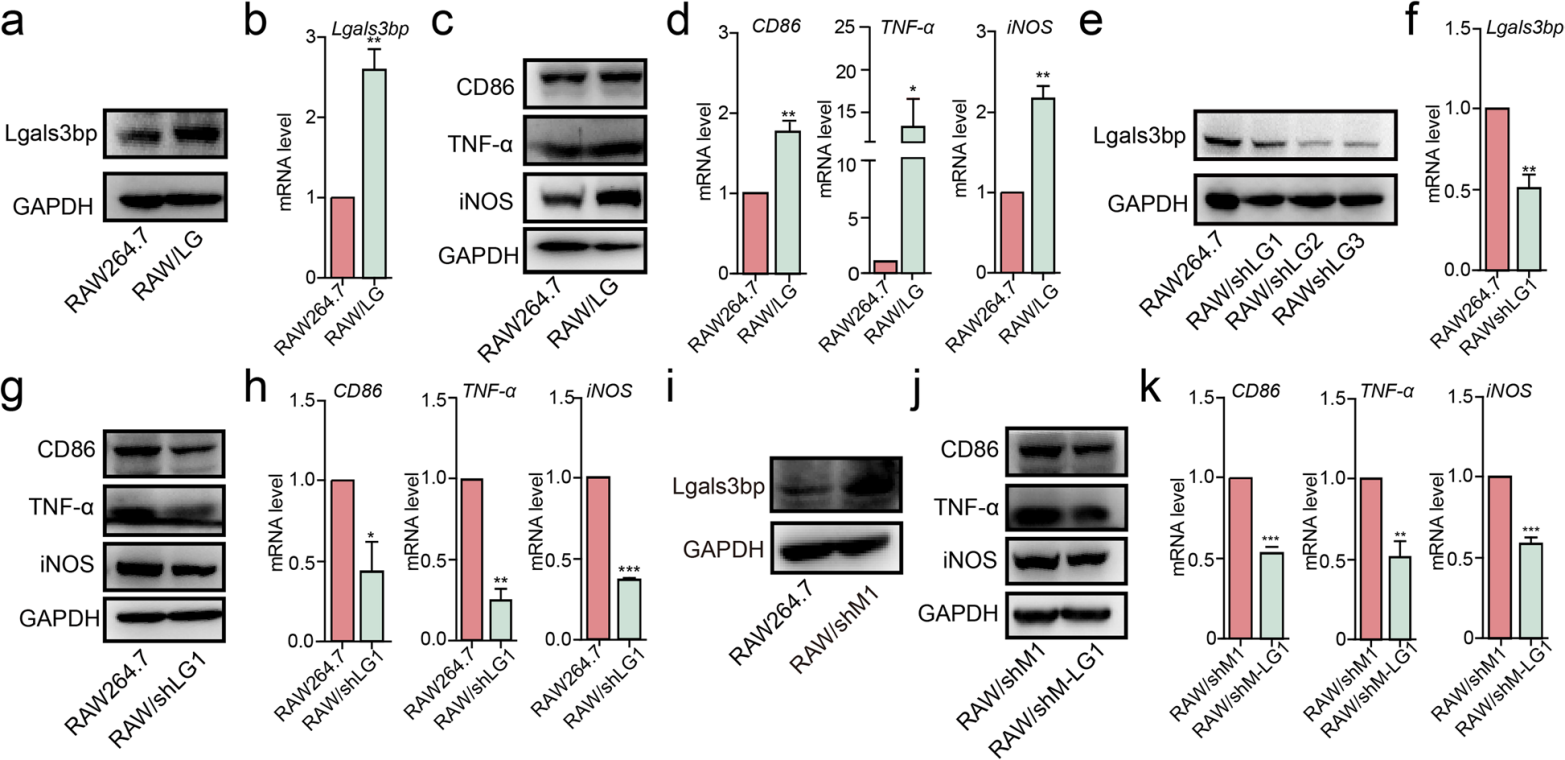
Effects of Lgals3bp on polarization. Lgals3bp was overexpressed (RAW/LG) or silenced (RAW/shLG1/2/3) in RAW264.7 cells. Target genes and proteins were analyzed by qRT-PCR or western blotting, respectively. **a**, **b** Lgals3bp expression in RAW/LG cells. **c**, **d** Expression of TNF-α, CD86 and iNOS in RAW/LG cells. **e**, **f** Lgals3bp expression in RAW/shLG1 cells. **g**, **h** Expression of TNF-α, CD86 and iNOS in RAW/shLG1 cells. (**i**) Lgals3bp expression in RAW/shM1 cells. **j**, **k** Expression of TNF-α, CD86 and iNOS in RAW/shM1 cells silenced Lgals3bp (RAW/shM-LG1)

Administration of exogenous Lgals3bp has been demonstrated to stimulate macrophages to release a multitude of proinflammatory cytokines, encompassing IL6, TNF-α and IL1β [39, 40]. These cytokines are well-known markers intimately linked with the polarization of macrophages towards the M1 phenotype [41]. Upon overexpression of Lglas3bp in RAW264.7 cells (RAW/LG) (Fig. 4a, b), we observed an elevation in the expression of M1 markers, including CD86, TNF-α and iNOS (Fig. 4c, d, S4a). Conversely, upon silencing of Lglas3bp expression in RAW264.7 cells (RAW/shLG1/2/3) (Fig. 4e, f), there was a reduction in the levels of these indicative markers (Fig. 4g, h, S4b). Interestingly, RAW/shM1 cells, which already exhibit an M1-like phenotype, exhibited an increase in Lgals3bp expression (Fig. 4i). Silencing Lgals3bp in these cells (RAW/shM-LG1) (Fig. S4c, d) led to the suppression of key indicative markers of the M1 phenotype (Fig. j, k). These results suggest that Lgals3bp may play a role in facilitating the transition from M0 to M1 polarization.

### Effect of bisecting GlcNAc on Lgals3bp exprssion

**Fig. 5:**
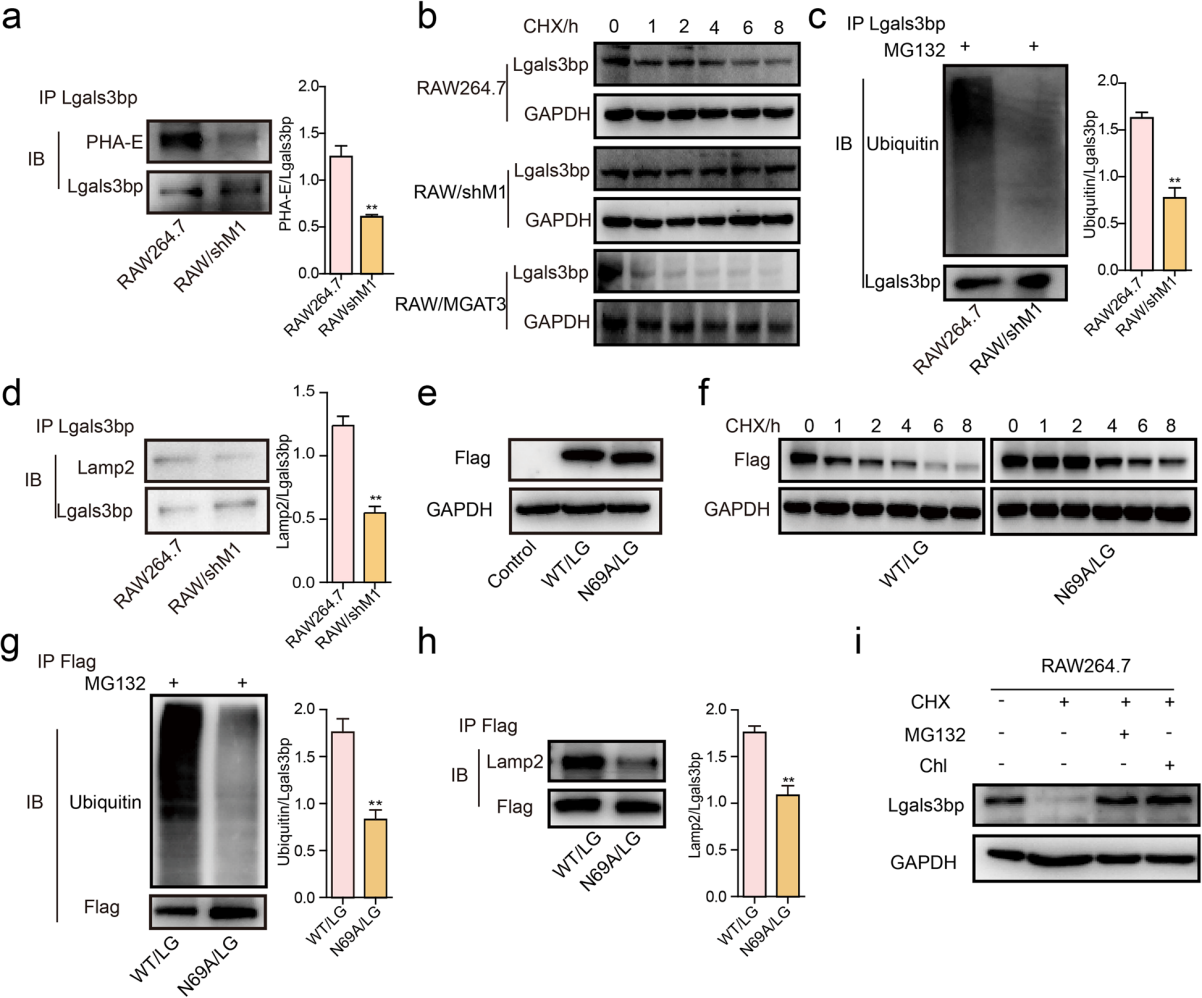
The function of bisecting GlcNAc on Lgals3bp. **a** Bisecting GlcNAc modification on Lgals3bp in RAW/shM1 cells by IP. **b** The Lgals3bp expression after CHX (150 µg/mL) treatment for indicated times was detected by western blotting. **c** The ubiquitination of Lgals3bp after MG132 (5 μM) treatment for 12 h in RAW264.7/shM1 cells was detected by Co-IP/western blotting. **d** Interaction of Lamp2 and Lgals3bp in RAW264.7/shM1 cells was detected by Co-IP/western blotting. **e** Flag-tagged wild type (WT/LG) or deglycosyl mutant at position 69 (N69A/LG) of Lgals3bp was transfected into HEK 293 T cells. **f** The expression of WT or N69A mutant of Lgals3bp was detected by western blotting after treatment with CHX (150 µg/mL) for the indicated times. **g** The ubiquitination of WT or N69A mutant of Lgals3bp was detected by Co-IP/western blotting after treatment with MG132 (5 μM) for 12 h in HEK 293 T cells. **h** The interaction between Lamp2 and WT or N69A mutant of Lgals3bp in HEK 293 T cells was detected by Co-IP/western blotting. **i** RAW264.7 cells were treated with CHX and MG132 or Chl for 12 h, then Lgals3bp expression were detected by western blotting

Based on the increased expression of Lgals3bp (Fig. 4i) accompanied with the decrease in its bisecting GlcNAc modification after silencing MGAT3 (Fig. 5a), we hypothesized that the stability of Lgals3bp is affected by bisecting GlcNAc. To test this hypothesis, we treated RAW/shM1 cells with cycloheximide (CHX), a protein synthesis inhibitor, to assess the degradation of Lgals3bp. We found that RAW/shM1 cells exhibited a decelerated degradation of Lgals3bp compared to control group. Conversely, RAW/MGAT3 cells showed an accelerated degradation of Lgals3bp (Fig. 5b). When treated with MG132, the proteasome inhibitor, Lgals3bp ubiquitination was inhibited under impairing bisecting GlcNAc in RAW/shM1 cells (Fig. 5c). Impairing bisecting GlcNAc in RAW/shM1 cells also resulted in less interaction between Lgals3bp and the lysosomal protein Lamp2 (Fig. 5d). Additionally, upon overexpression of Lgals3bp with a Flag-tag (WT/LG) or its deglycosyl mutant at position 69 (N69A/LG) in HEK 293 T cells (Fig. 5e), we observed that the N69A/LG mutant exhibited greater stability compared to the WT/LG, along with reduced ubiquitination modification and less interaction with Lamp2 (Fig. 5f-h). MG132 or the lysosomal inhibitor chloroquine (Chl) both restored the Lgals3bp expression (Fig. 5i), indicating that bisecting GlcNAc facilitated Lgals3bp degradation through proteasomal and lysosomal pathways. These results provide insights into the mechanisms of bisecting GlcNAc underlying the regulation of Lgals3bp stability and its potential implications in macrophage polarization and function.

### Mechanism of Lgals3bp and its bisecting GlcNAc modification in M1 polarization

**Fig. 6:**
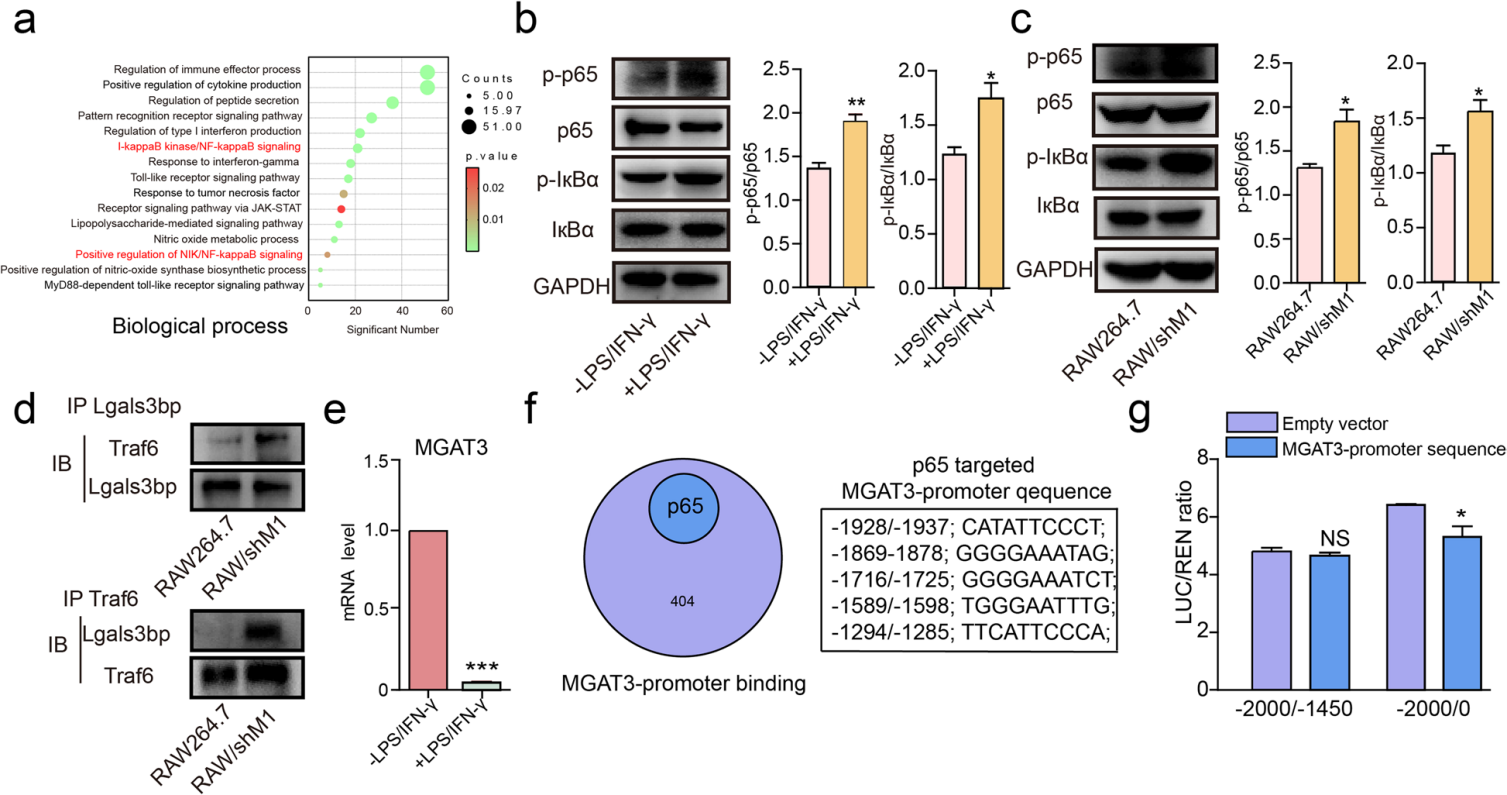
The correlation of NF-κB, Lgals3bp, and MGAT3. **a** GO analysis of differentially expressed proteins in M0-M1 polarization of RAW264.7 cells. **b**, **c** Expression of p65, p-p65, IкBα and p-IкBα in RAW264.7 cells stimulated by LPS and IFN-γ (**b**) or RAW/shM1 cells (**c**) by western blotting. **d** Interaction between Lgals3bp and Traf6 in RAW/shM1 cells by Co-IP. **e** MGAT3 mRNA level in RAW264.7 cells stimulated by LPS and IFN-γ. **f** PROMO analysis to predict the transcription factors that bind to the MGAT3 promoter. **g** Dual-luciferase reporter assays in HEK 293 T cells transfected with p65 and the MGAT3 promoter sequence

With the proteomics data, we found that the main biology processes during the M0-M1 polarization were involved in the NF-кB pathway, cytokine production pathway, and Toll-like receptor signaling pathway (Fig. 6a, S5a). We observed an upregulation of phosphorylated p65 (p-p65) and phosphorylated IкBα (p-IкBα) in response to LPS/IFN-γ or silencing of MGAT3 in RAW264 cells (Fig. 6b, c). Furthermore, overexpression of Lgals3bp presented the upregulation of p-p65 and p-IкBα, while silence of Lgals3bp lead to their inactivation (Fig. S5b, c). Prior studies have suggested that the interaction between Lgals3bp and Traf6 can influence the signaling cascade of the NF-κB pathway [40]. In our study, we found that Lgals3bp with reduced levels of bisecting GlcNAc exhibited a stronger binding affinity to Traf6 (Fig. 6d). Taken together, these findings indicate that the absence of bisecting GlcNAc on Lgals3bp can alter the interaction between Lgals3bp and Traf6, leading to the activation of the NF-кB pathway and ultimately driving M1 polarization.

Based on M0-M1 polarization in RAW264.7 cells, we observed a decreased MGAT3 in the protein expression (Fig. 1e). We further discovered the down-regulated mRNA level of MGAT3 (Fig. 6e). In addition, Lgals3bp showed a negative correlation with MGAT3 mRNA level (Fig. S5d), as well as the expression of MGAT3 and the level of bisecting GlcNAc. (Fig. S5e). To investigate the modulation mechanism of MGAT3 at transcription level, we performed PROMO analysis [42, 43] and predicted NF-кB/p65 as a transcription factor that binds to the MGAT3 promoter (Fig. 6f). Dual-luciferase reporter assays confirmed that p65 binds to the promoter region of MGAT3 from -1450 to -0 bp, and inhibits the transcriptional of MGAT3 (Fig. 6g). To further validate the role of p65 in regulating MGAT3 expression, we treated RAW264.7 cells with SC75741, an inhibitor of p65. We observed that the mRNA level of MGAT3 was increased upon treatment with SC75741 (Fig. S5f), along with an increase in MGAT3 protein expression and bisecting GlcNAc level (Fig. S5g).

## Discussion

There is growing evidence suggesting that changes in glycosylation can affect macrophage polarization and immune functionality. Glycosylation, an essential post-translational modification on proteins, plays a crucial role in cell metastasis, migration, and adhesion [44]. In glycoproteins, the glycan chain is typically connected to the peptide backbone through nitrogen (N-glycan) of asparagine or oxygen (O-glycan) of serine or threonine [45]. Bisecting GlcNAc is a type of glycan structure that suppresses the terminal modification of N-glycan [46]. This structure has been reported to possess immune suppression functions and can impact the phagocytic activity of human monocyte-derived macrophages [47]. Yang et al. show that M2 macrophages possess elevated levels of bisecting GlcNAc [18], while immune cells in general exhibit minimal MGAT3 activity [48]. Additionally, M2 macrophages exhibit increased levels of bisecting GlcNAc compared to M0/M1 macrophages, suggesting a close relationship between bisecting GlcNAc and macrophage polarization [27]. In this study, we observed a significant reduction in bisecting GlcNAc during M0-M1 polarization, and impaired bisecting GlcNAc was found to drive M0-M1 polarization. Combining with previous findings, it is reasonable to believe that the bisecting GlcNAc modification plays a distinct role in influencing macrophage polarization.

Lgals3bp is a highly glycosylated protein involved in tumor growth and progression [49]. Through a glycoproteomics approach, we identified Lgals3bp as a protein carrying bisecting GlcNAc modifications. Lgals3bp is abundantly expressed after M1 polarization, and it has been identified as an inflammatory marker of macrophages [50]. It is also positively expressed on tissue-resident macrophages, such as alveolar macrophages and Kupffer cells [51]. Upregulation of Lgals3bp promotes macrophage polarization towards the M1 phenotype. Lgals3bp secreted from osteosarcoma binds the Lgals3 ligand on the surface of M1 macrophage, thereby enhancing their anti-tumor abilities [52]. Previous reports have indicated that the removal of both N-glycosylation and O-glycosylation from Lgals3bp impairs its secretion [53, 54]. More recently, the presence of bisecting GlcNAc structures has been found to be closely associated with the polarization of M1 and M2 macrophages [27], suggesting a potential role of bisecting GlcNAc modification in the biological effect of Lgals3bp. In this study, we discovered that Lgals3bp with low levels of bisecting GlcNAc and its deglycosyl mutant of Lgals3bp at position 69 are both less prone to degradation. Similar findings regarding the impact of bisecting GlcNAc on protein stabilization have been reported in several recent studies [31, 44]. These results suggest a positive relationship between Lgals3bp and M1 macrophages, and the stability of intracellular Lgals3bp is influenced by bisecting GlcNAc.

Lgals3bp is commonly enriched in extracellular vesicles from tumor cells, and its serum level has been shown to be a prognostic indicator in various cancers [49, 55]. When secreted as soluble Lgals3bp, it can interact with various surface proteins, including Lgals3 [52], integrins [56] and Siglecs [57], to exert its effects, particularly in innate immune. In the case of viral infection, Lgals3bp forms complex with Traf6 or Traf3, and subsequently recruits TAK1 and TBK1, which then signal the translocation of the transcription factors NF-κB, IRF3, and IRF7 from the cytoplasm to the nucleus. This triggers the production of IFNs and inflammatory cytokines [40]. In this study, we observed that the interaction between Lgals3bp and Traf6 was strengthened when the bisecting GlcNAc modification on macrophages was reduced, resulting in the activation of the NF-κB signaling pathway. These findings collectively suggest the role of NF-κB in mediating the effect of the Lgals3bp-Traf6 complex on macrophage polarization.

**Fig. 7:**
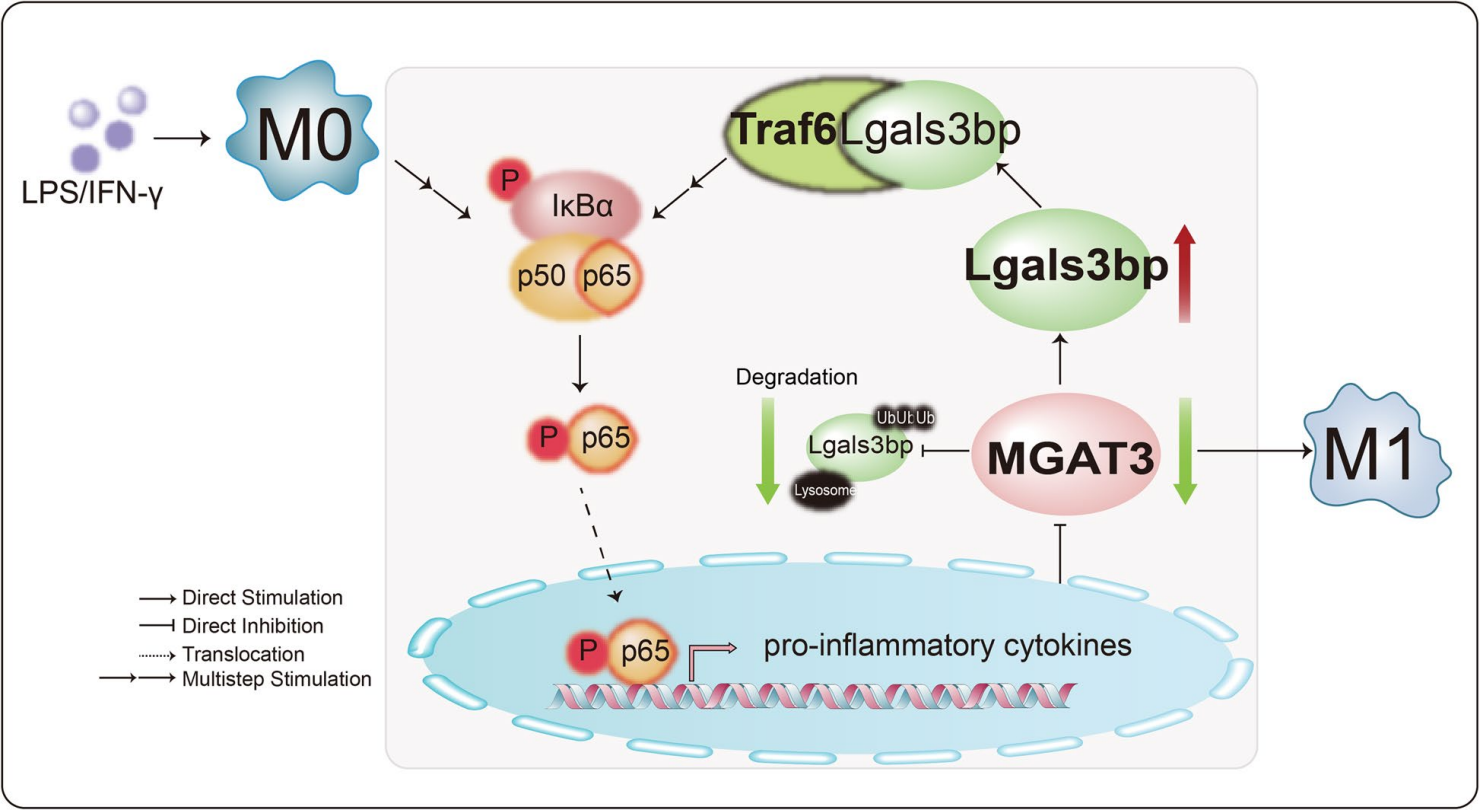
Conceptual model of bisecting GlcNAc mediating M1 polarization of macrophage

Since MGAT3 expression was down-regulated at the mRNA level, we hypothesized that the NF-κB/p65 signaling pathway might serve as a crucial regulator of MGAT3. In the active NF-κB pathway, the IκB kinase (IKK) complex phosphorylates IκBs, leading to the degradation of IkBs and translocation of NF-κB/p65 into the nucleus [58]. The p65 subunit, also named RelA, has been identified as transcription factor for M1-related proinflammatory genes [59]. Combined with PROMO analysis and dual-luciferase assay, we confirmed that p65 acts as a transcription inhibitor of MGAT3. Based on these findings, we propose that the reduction in bisecting GlcNAc could drive M0-M1 polarization by stabilizing Lgals3bp. In turn, stabilized Lgals3bp promotes M1 polarization by activating the NF-кB pathway. Concurrently, the activation of the NF-кB pathway significantly suppresses the transcription of MGAT3, resulting in reduced levels of bisecting GlcNAc modification on Lgals3bp (Fig. 7).

## Conclusion

In summary, our study emphasizes the significance of glycosylation in macrophage polarization and highlights the potential for modifying macrophages through alterations in glycosylation.

### Supplementary Information

**Additional file 1. **

**Additional file 2. **

## Data Availability

All data contained within this article are available from the corresponding authors upon reasonable request.

## Abbreviations

LPS: Lipopolysaccharide
TAMs: Tumor-associated macrophages
OGT: O-GlcNAc transferase
CM: Conditional medium
PMs: Peritoneal macrophages
BMMCs: Bone marrow mononuclear cells
Lgals3bp: Galectin 3 binding protein
