# Supplementary material for: Impaired bisecting GlcNAc reprogrammed M1 polarization of macrophage
Source: Cell Commun Signal. 2024 Jan 26;22:73. doi: 10.1186/s12964-023-01432-6 (PMC10811823; doi:10.1186/s12964-023-01432-6)
Supplement: Supplementary file 2 — Additional file 1. [file 12964_2023_1432_MOESM1_ESM.docx]

**Supplementary Information**

**Impaired Bisecting GlcNAc Reprogrammed M1 Polarization of Macrophage**

Xin He^1^, Bowen Wang^1^, Wenli Deng^1^, Jinhua Cao^1^, Zengqi Tan^1^, Xiang Li^2*^, Feng Guan^1,*^

1. Key Laboratory of Resource Biology and Biotechnology Western China, Ministry of Education; Provincial Key Laboratory of Biotechnology, College of Life Sciences, Northwest University, Xi'an, 710069, China.

2. Institute of Hematology, School of Medicine, Northwest University, Xi’an, 710069, China

Correspondence to Xiang Li (e-mail: xiangli@nwu.edu.cn) or Feng Guan (e-mail: guanfeng@nwu.edu.cn), Tel: +86-29-88303534, College of Life Sciences, Northwest University, No, 229, Taibai North Road, Xi’an, Shaanxi 710069, China

**Methods**

Flow Cytometry

Flow cytometry was performed following the protocol described previously ^1^. Briefly, cells were fixed in 4% paraformaldehyde (Sigma-Aldrich; St. Louis, MO, USA) at room temperature (RT) for 15 min, and then incubated with primary antibody (Table S1) at RT for 30 min. After washing twice with PBS, cells were incubated with appropriate fluorescent-conjugated secondary antibody (Beyotime) at RT for 30 min in the dark and then analyzed using flow cytometry (FACS; ACEA NovoCyte; Hangzhou, China).

For cell proliferation analysis, the iClikTM EDU Andy Fluor 657 Flow Cytometry Assay Kit (cat #A008, Keygen; Jiangsu, China) was used following the manufacturer’s instructions. To assess cell apoptosis, cells were resuspended in 100 μL binding buffer with 5 μL Annexin V (BioLegend; San Diego, CA, USA) and 5 μL 7-AAD (BioLegend) for 30 min at 4℃. The reaction was then stopped by adding 400 μL binding buffer. FACS was employed in the cell proliferation and apoptosis assay.

Immunofluorescence

Immunofluorescence was performed following the protocol described previously ^2^. Briefly, cells were fixed with 4% paraformaldehyde (Sigma-Aldrich) in the confocal dish at RT for 15 min, permeabilized with 0.2% Triton X-100 at RT for 10 min, and then blocked with 3% BSA (Beyotime) at 37℃ for 30 min. Cells were incubated with the primary antibody (Table S2) at 4℃ overnight, and stained with the appropriate fluorescent-conjugated secondary antibody (Beyotime) at RT for 1 h. Then, cells were incubated with DAPI (Beyotime) at RT for 15 min. Finally, the fluorescence images were visualized using confocal microscope (Olympus FN1000; Olympus; Tokyo, Japan).

Quantitative Real-time Polymerase Chain Reaction (qRT-PCR)

Total RNA was extracted using TRIeasy™ LS Total RNA Extraction Reagent (YEASEN; Tianjin, China) as described in the manufacturer’s manual. HiScript II Q RT SuperMix (cat # R223-01, Vazyme; Nanjing, China) was used for first-strand cDNA synthesis. qRT-PCR was performed with AceQ qPCR SYBR Green Master Mix (cat # Q111-02, Vazyme). Primers are listed in Table S2, and gene expression was analyzed by 2^−ΔΔCt^ method.

Western blotting

Cells were lysed in radioimmunoprecipitation assay (RIPA) buffer (50 mM Tris, pH 7.2, 1% Triton X-100, 0.5% sodium deoxycholate, 0.1% SDS, 150 mM NaCl, 10 mM MgCl_2_ and 5% glycerol) containing 1% proteinase inhibitors (Sigma-Aldrich) and 1% phosphatase inhibitors (Sigma-Aldrich). Protein concentration was measured using the BCA Protein Assay Kit (#P0011, Beyotime). The proteins (25 μg) were then separated using SDS-PAGE and transferred onto polyvinylidene difluoride (PVDF) membranes (Bio-Rad; Hercules, CA, USA). Membranes were blocked with 3% BSA in TBST at 37℃ for 1 h. The incubation of primary antibodies (Table S2) and HRP-conjugated secondary antibody (Beyotime) was performed as mentioned in the immunofluorescence protocol. Membranes were visualized using enhanced chemiluminescence reagent (ECL; Vazyme Biotech; Nanjing, China) and imaged using chemiluminescent imaging system (Tanon; Shanghai, China).

Lectin blotting

Lectin blot was performed following the protocol of western blotting. Briefly, the biotin-conjugated PHA-E was used for detecting the level of bisecting GlcNAc modification (Table S1). Subsequently, membranes were incubated with the VECTASTAIN Elite ABC kit (cat # KT6100, Vector Laboratories) at RT for 1 h.

Plasmid construction and cell transfection

Genes were amplified via PCR and then linked to the lentiviral vector pLVX-AcGFP-N1 (Takara; Shiga, Japan). Short hairpin RNAs (shRNAs) targeting MGAT3 or Lgals3bp (Table S2) were linked to the lentiviral vector pLKO.1-puro (Takara). The constructed lentiviral vector, pMD2.G and psPAX2 (Addgene; Cambridege, MA, USA) were co-transfected into HEK293T. After 48 h, lentivirus particles were collected and transfected into target cells. The transfected cells were selected using puromycin for 3 days and confirmed using western blotting.

Transwell assay

Cells were transferred to the upper chamber of inserts with 8 μm pores (Corning; Cambridge, MA, USA). The bottom chamber was filled with complete medium. After 24 h, cells migrating across the upper chamber membrane were stained with 0.1% crystal violet (Beyotime) and visualized under a microscope (Sunnyoptical; Ningbo, China).

Immunohistochemistry

The tissue was manufactured to be the formalin-fixed paraffin embedded (FFPE) slide as described previously ^3^. Slides were incubated with Ki67 (Table S2), and then stained using the DAB Horseradish Peroxidase Color Development Kit (#P0203, Beyotime). Slides were photographed using a glass slide scanning imaging system (Teksqry; Shenzhen, China).

Immunoprecipitation (IP) /Co-Immunoprecipitation (Co-IP)

IP/Co-IP was performed following the protocol described previously ^4^. Briefly, primary antibody (2 μg) was added into protein lysates (0.5 mg) at 4℃ for 1 h. Protein A+G Agarose (20 μL) (Beyotime) was added and then incubated at 4℃ overnight. The samples were denatured using SDS sample loading buffer, and analyzed by western blotting.

Dual-Luciferase reporter assay

Promoter sequences were amplified from the whole genome of RAW264.7 via PCR and linked to luciferase reporter vector pGL3. Basic (#E1751, Promega; Madison, WI, USA). p65 was inserted into lentiviral vector pLVX-AcGFP-N1. Two constructed plasmids and pRL-TK (#D2760, Beyotime) vector were co-transfected into HEK 293T cells. Relative luciferase activity was determined as per manufacturer's instructions of Dual-Luciferase Reporter Gene Assay Kit (#RG027, Beyotime).

**Table S1 Antibodies**

**
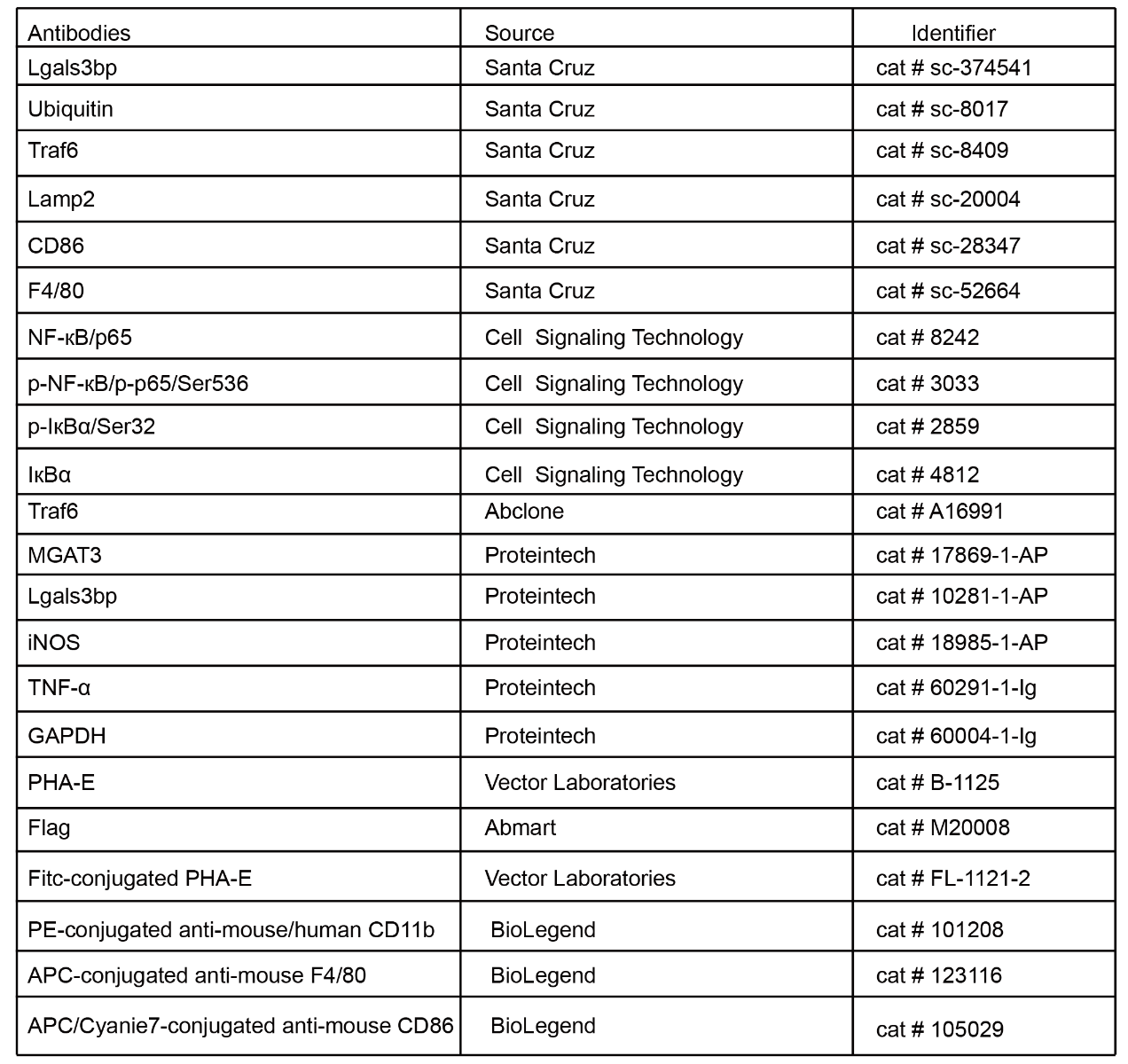
**

**Table S2 shRNAs and Primers**


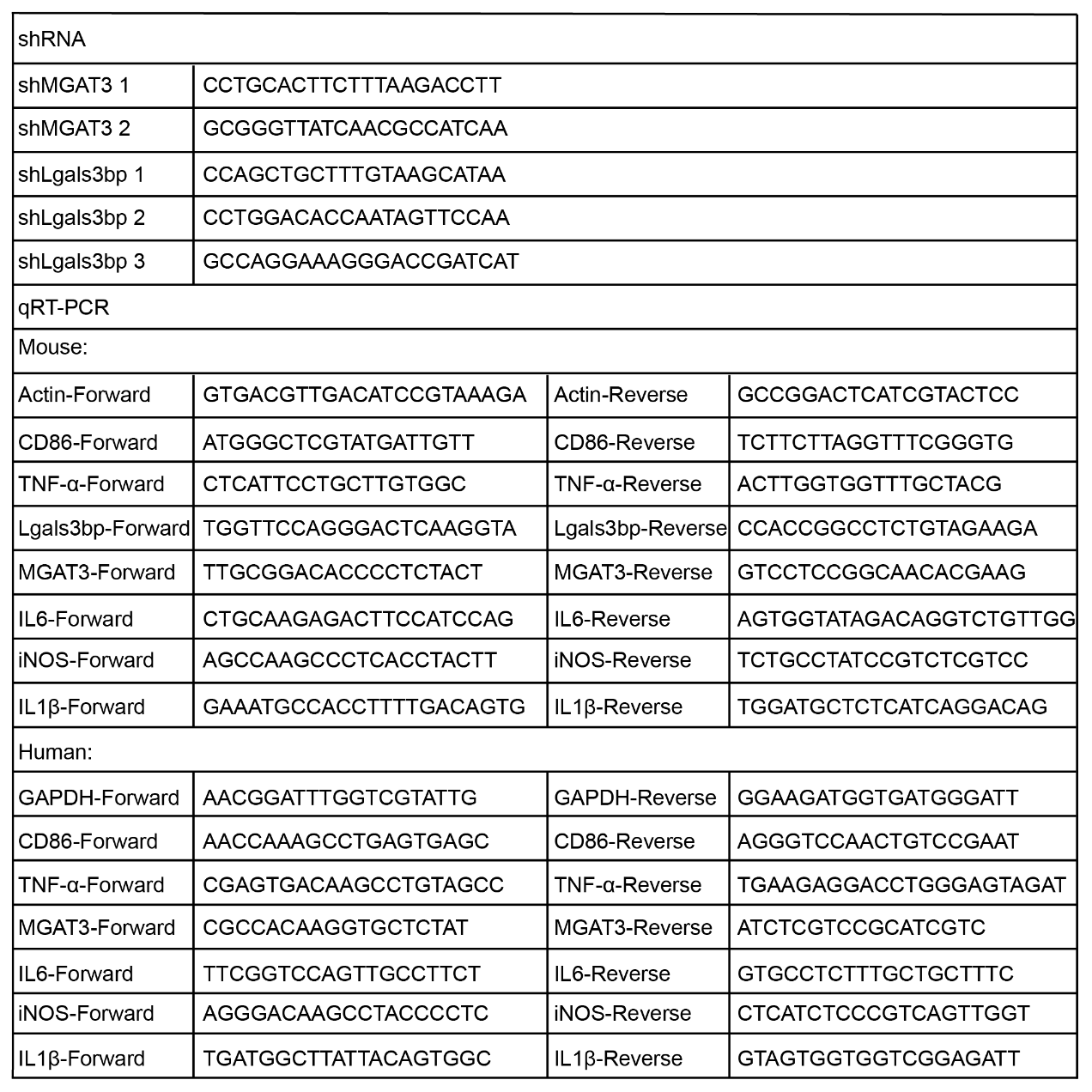


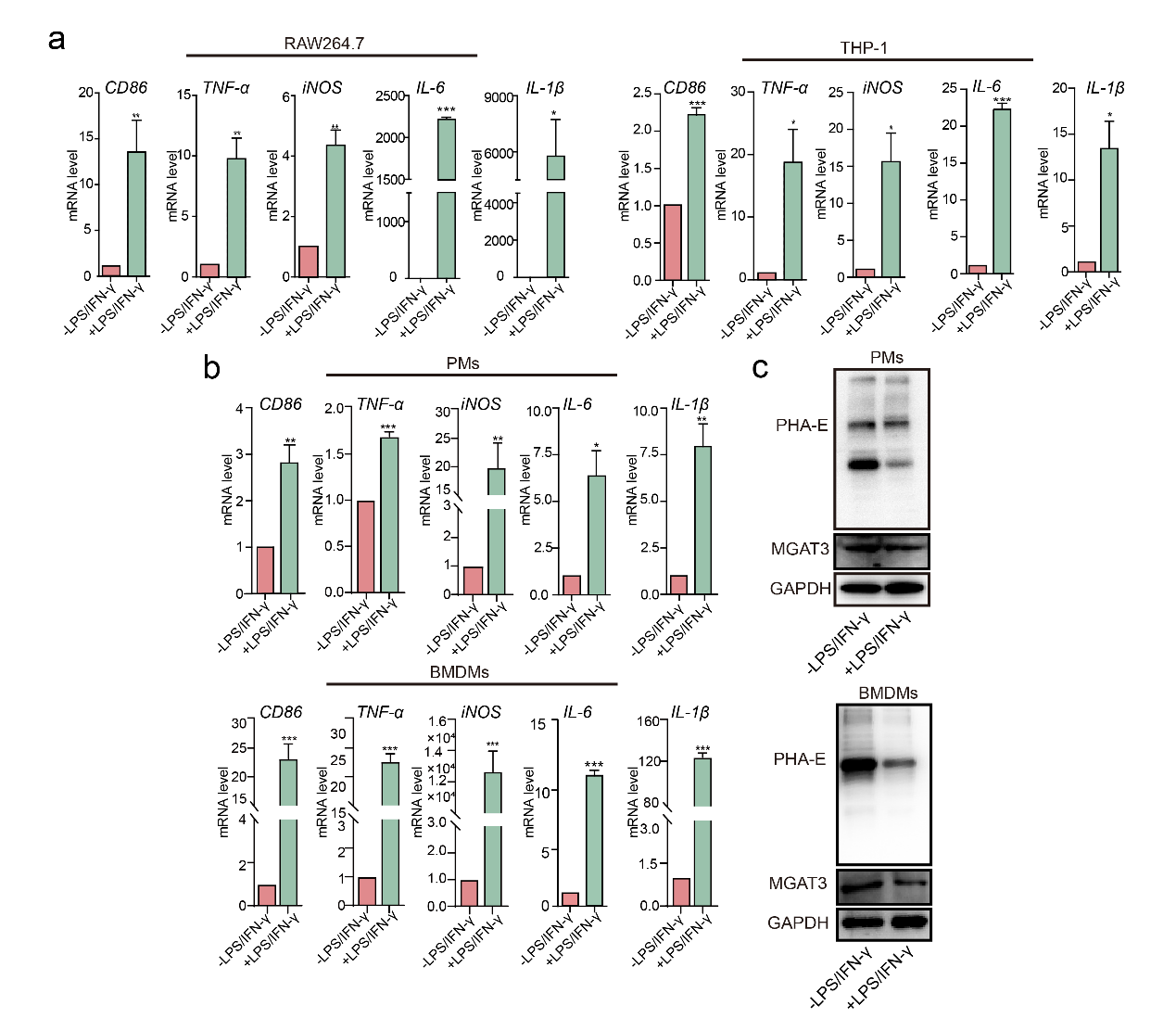


**Supplementary Fig. 1 M0-M1 polarization of RAW264.7, THP-1, PMs and BMDMs**

(**a-c**) The mRNA level of CD86, TNF-α, iNOS, IL6 and IL1β by qRT-PCR in RAW264.7/THP-1 cells (**a**) or PMs/BMDMs (**b**). Bisecting GlcNAc level and MGAT3 expression in PMs and BMDMs evaluated by lectin/western blotting **(c)**.


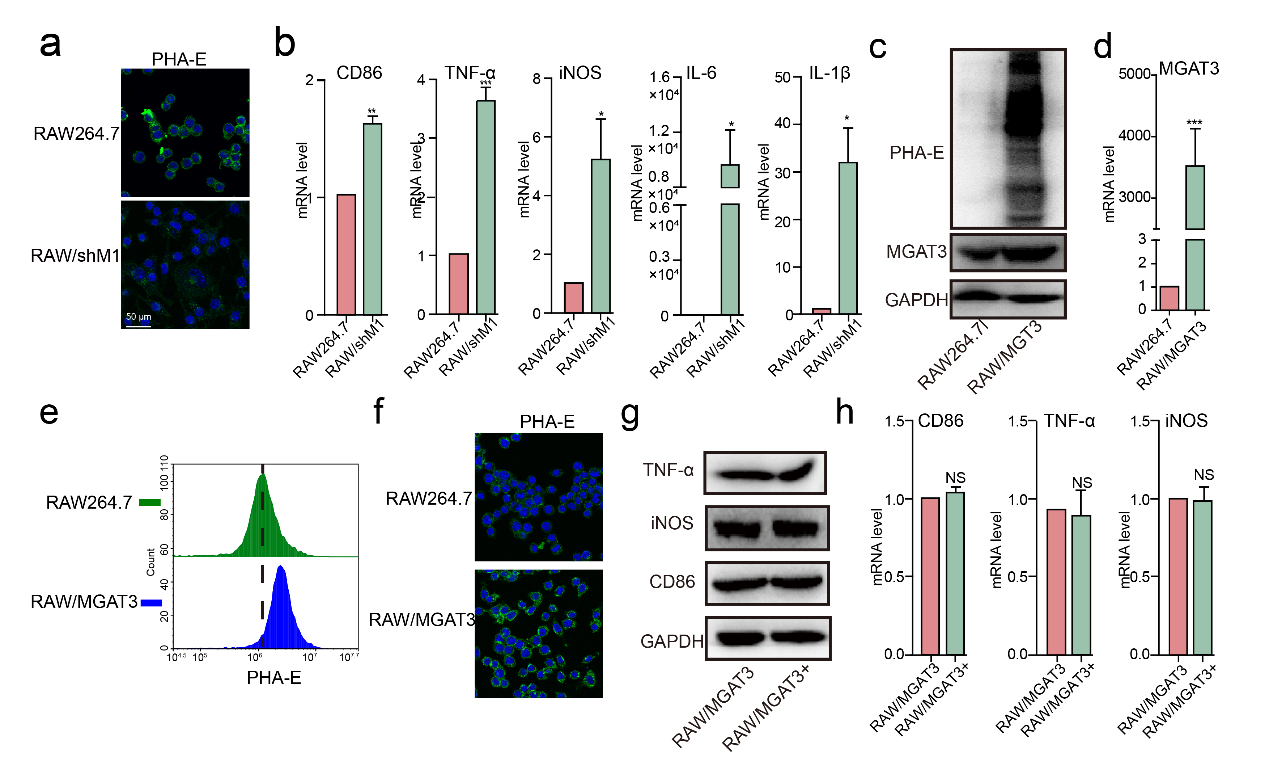


**Supplementary Fig. 2 Effect of inhibiting or enhancing bisecting GlcNAc modification on M1 polarization**

(**a**) Bisecting GlcNAc level in RAW/shM1 cells by immunofluorescence. (**b**) The mRNA level of CD86, TNF-α, iNOS, IL6 and IL1β by qRT-PCR in RAW/shM1 cells. (**c**) MGAT3 was forced in RAW264.7 cells (RAW/MGAT3), MGAT3 expression was determined by western blotting and bisecting GlcNAc level was evaluated by lectin blotting. (**d**) MGAT3 mRNA level in RAW/MGAT3 cells by qRT-PCR. (**e&f**) Bisecting GlcNAc level in RAW/MGAT3 cells by FACS (**e**) and immunofluorescence (**f**). (**g-h**) RAW/MGAT3 cells was stimulated with IFN‐γ and LPS. Expression of CD86, TNF-α and iNOS by western blotting (**g**) and qRT-PCR (**h**).


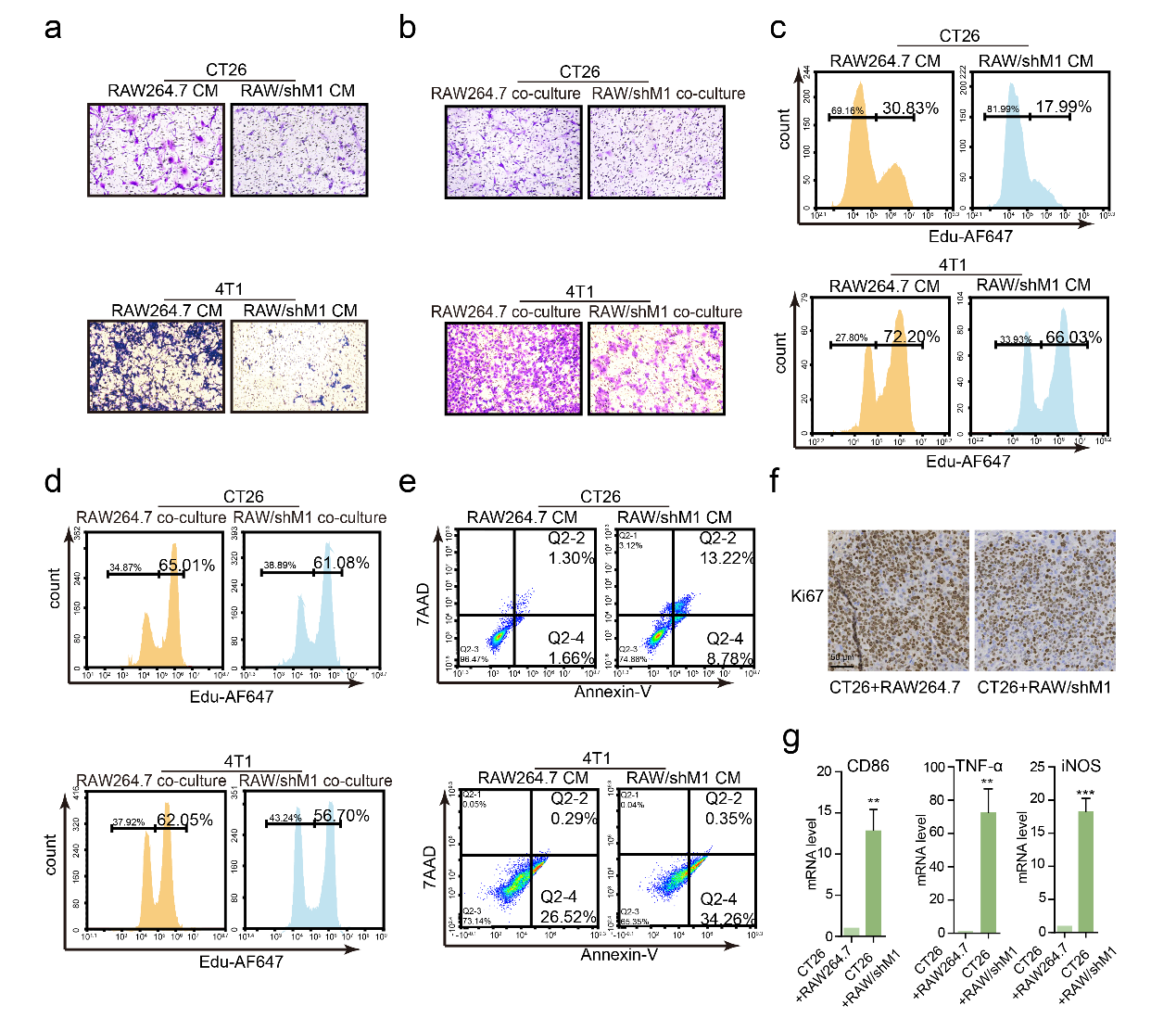


**Supplementary Fig. 3** **Effect of RAW/shM1 on tumor cells**

**(a)** CT26 and 4T1 cells were treated with conditional medium (CM) from RAW264.7 or RAW264.7/shM1 cells for 48 h, and then the migration of CT26 and 4T1 cells was analyzed. **(b)**

CT26 and 4T1 cells were incubated with RAW264.7 or RAW264.7/shM1 cells in the co-culture model, for 48 h, and then the migration of CT26 and 4T1 cells was analyzed. **(c&d)** Proliferation of CT26 and 4T1 cells after CM treatment **(c)** or incubated in co-culture model **d)**. **(e)** Apoptosis of CT26 and 4T1 cells after CM treatment. **(f)** Ki67 staining. (**g**) The mRNA level of CD86, TNF-α and iNOS on purified macrophages from tumor model.


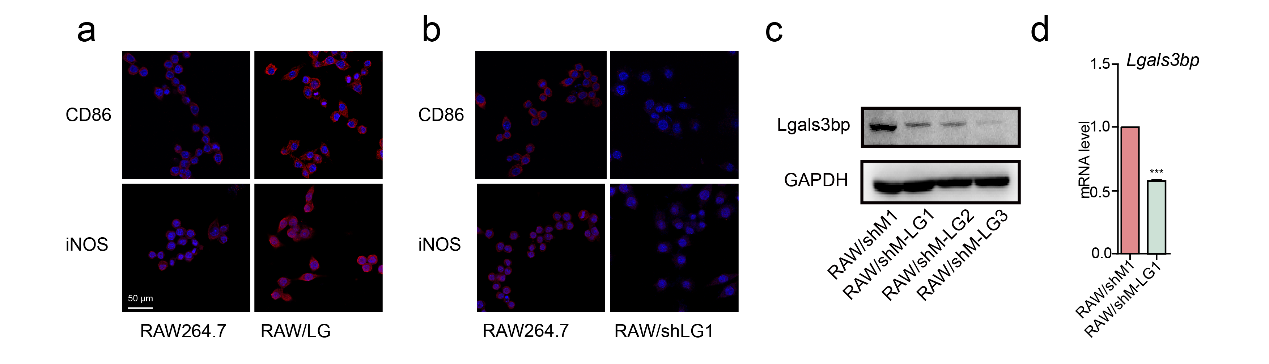


**Supplementary Fig. 4** **The relationship between Lgals3bp and M1 markers**

(**a**) Immunofluorescence of CD86 and iNOS in RAW/LG cells. (**b**) Immunofluorescence of CD86 and iNOS in RAW/shLG1 cells. (**c&d**) Lgals3bp expression in RAW/shM-LG1/2/3 cells confirmed by western blotting (**c**) and qRT-PCR (**d**).


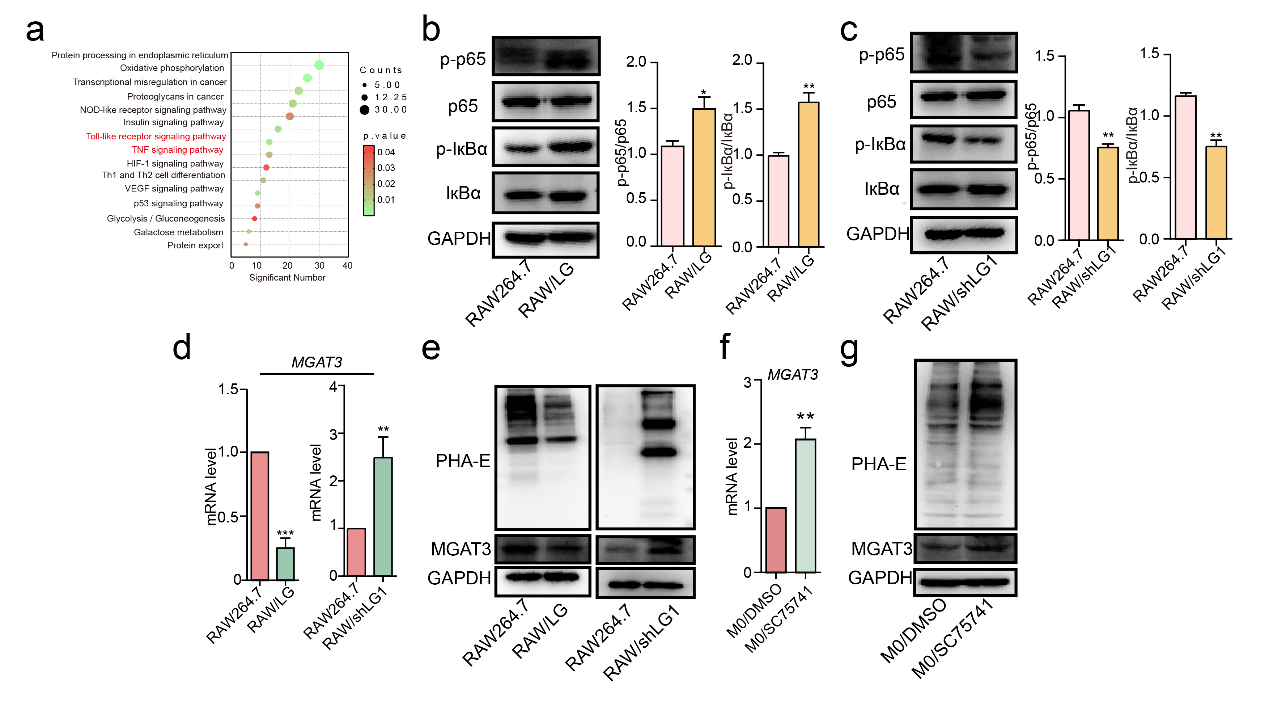


**Supplementary Fig. 5** **Effect of Lgals3bp on NF-кB signaling pathway** **and MGAT3 expression.**

(**a**) KEGG analysis in M0-M1 polarization of RAW264.7 cells. (**b-c**) Expression of p65, p-p65, IкBα and p-IкBα evaluated in RAW/LG cells (**b**) and RAW/shLG1 cells (**c**) by western blotting. (**d**) The mRNA expression of MGAT3 in RAW/LG and RAW/shLG1 cells by qRT-PCR. (**e**) Bisecting GlcNAc level and MGAT3 expression in RAW/LG and RAW/shLG1 cells by lectin/western blotting. (**f**) MGAT3 mRNA level after SC75741 (10 μM) treatment for 24 h in RAW264.7 cells by qRT-PCR. (**g**) Bisecting GlcNAc level and MGAT3 expression evaluated after SC75741 (10 μM) treatment for 24 h in RAW264.7 cells by lectin/western blotting.

**References**

1 Morrissey, S. M. *et al.*: **Tumor-derived exosomes drive immunosuppressive macrophages in a pre-metastatic niche through glycolytic dominant metabolic reprogramming**. *Cell Metab* 2021, **33**: 2040-2058.e2010.

2 Im, K., Mareninov, S., Diaz, M. F. P. & Yong, W. H.: **An Introduction to Performing Immunofluorescence Staining**. *Methods in molecular biology (Clifton, N.J.)* 2019, **1897**: 299-311.

3 Lim, A. S. & Lim, T. H.: **Fluorescence In Situ Hybridization on Tissue Sections**. *Methods in molecular biology (Clifton, N.J.)* 2017, **1541**: 119-125.

4 Tan, Z. *et al.*: **Bisecting GlcNAc modification diminishes the pro-metastatic functions of small extracellular vesicles from breast cancer cells**. *J Extracell Vesicles* 2020, **10**: e12005.
