# Supplementary figures and images for: Impaired bisecting GlcNAc reprogrammed M1 polarization of macrophage
Source: Cell Commun Signal. 2024 Jan 26;22:73. doi: 10.1186/s12964-023-01432-6 (PMC10811823; doi:10.1186/s12964-023-01432-6)

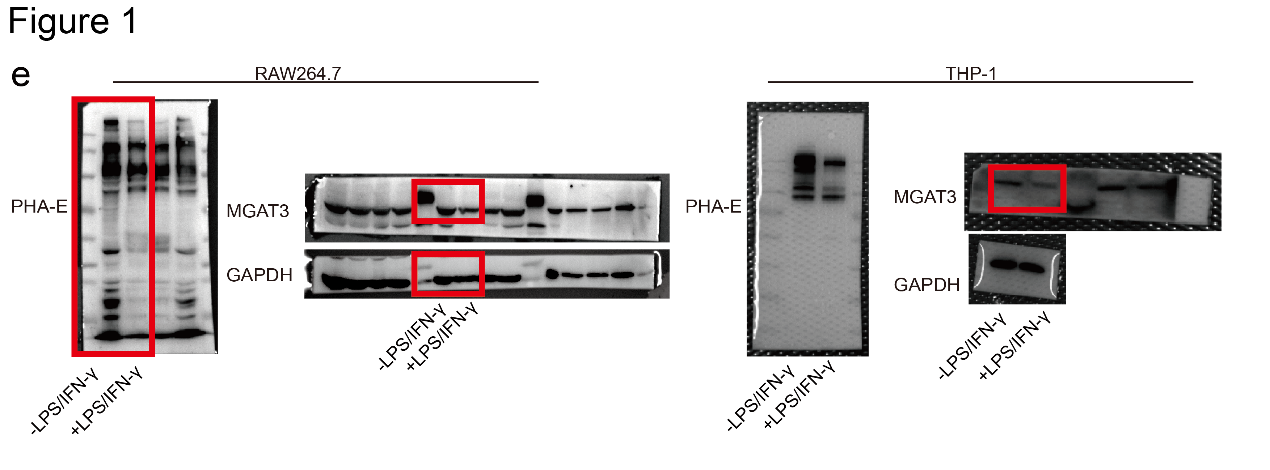


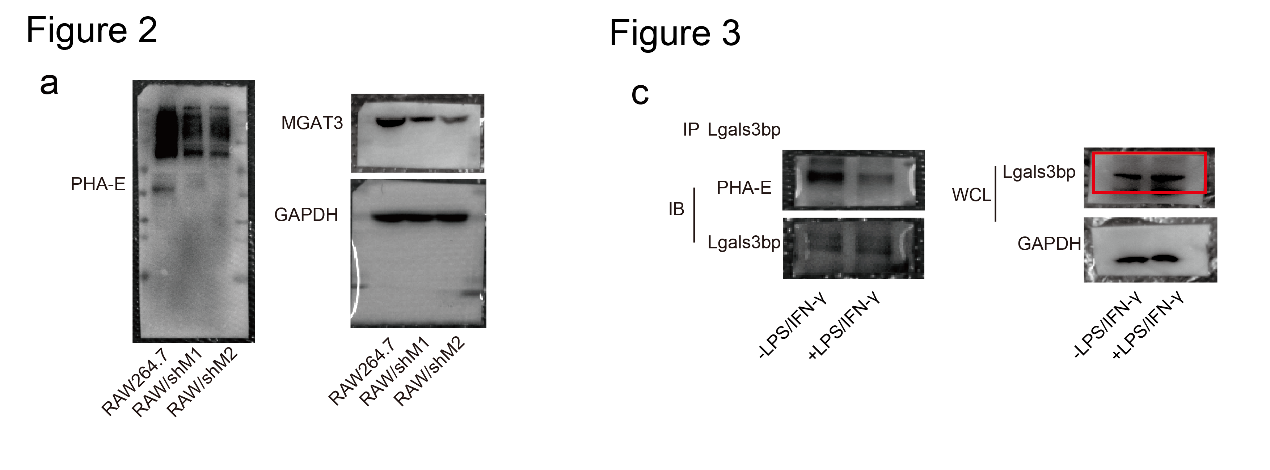


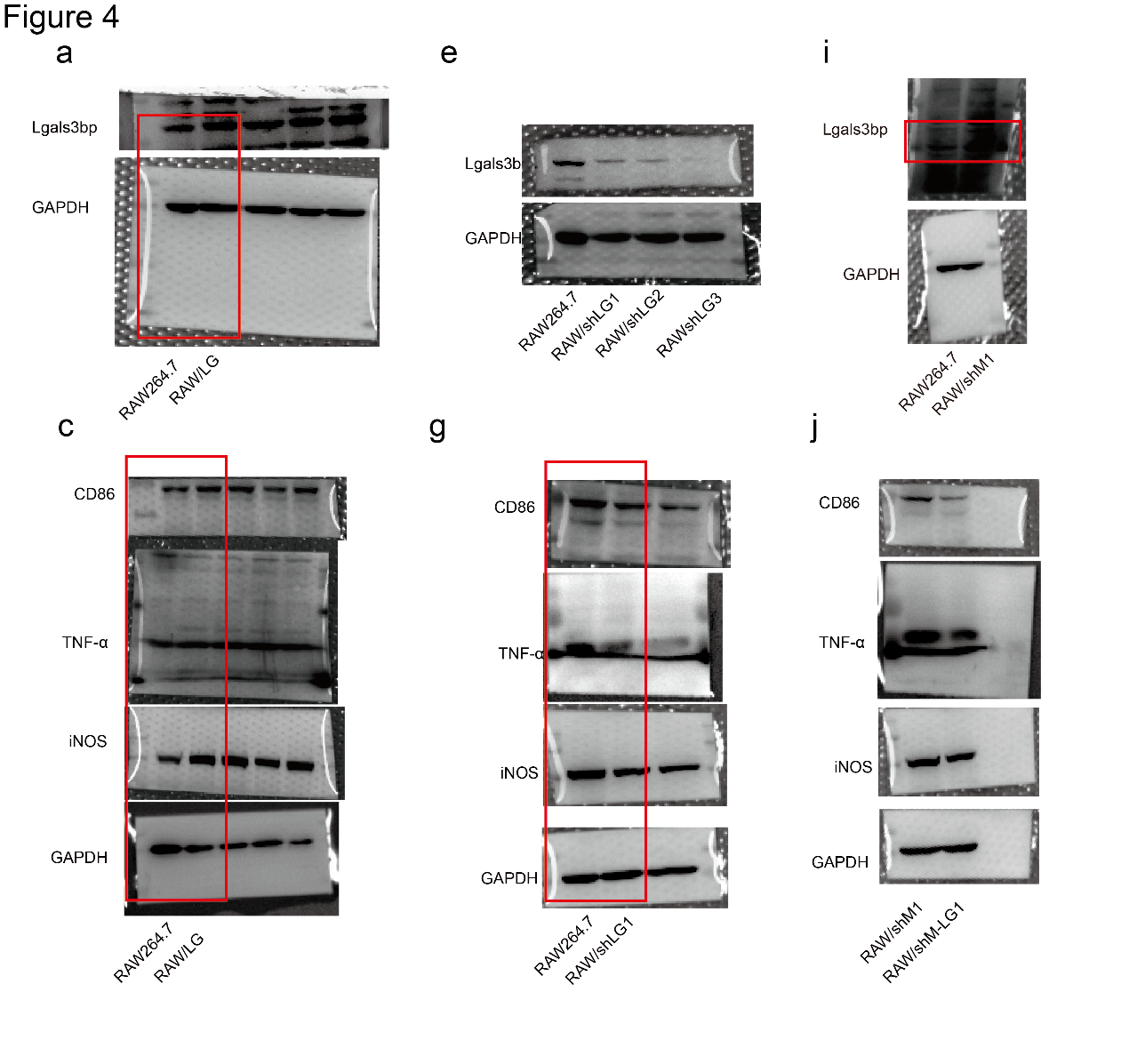


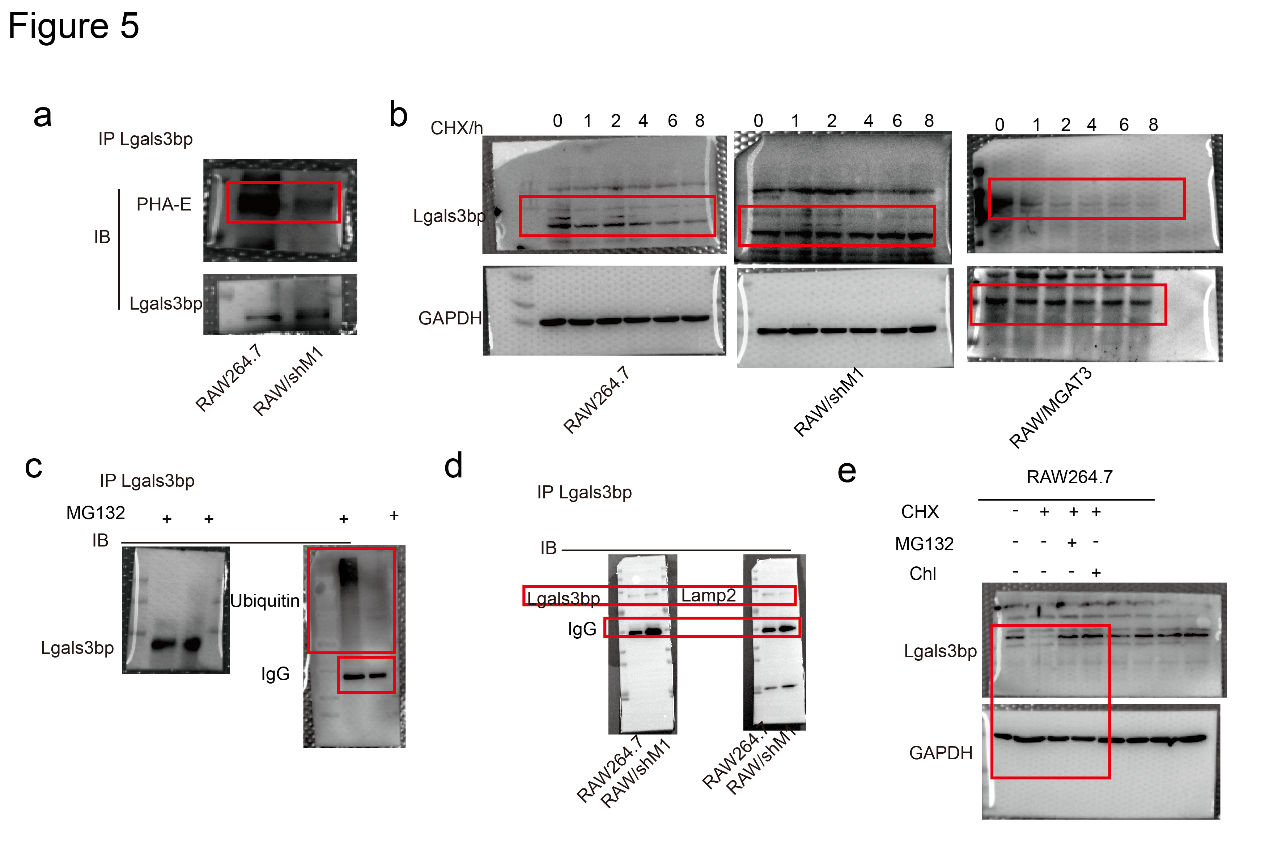


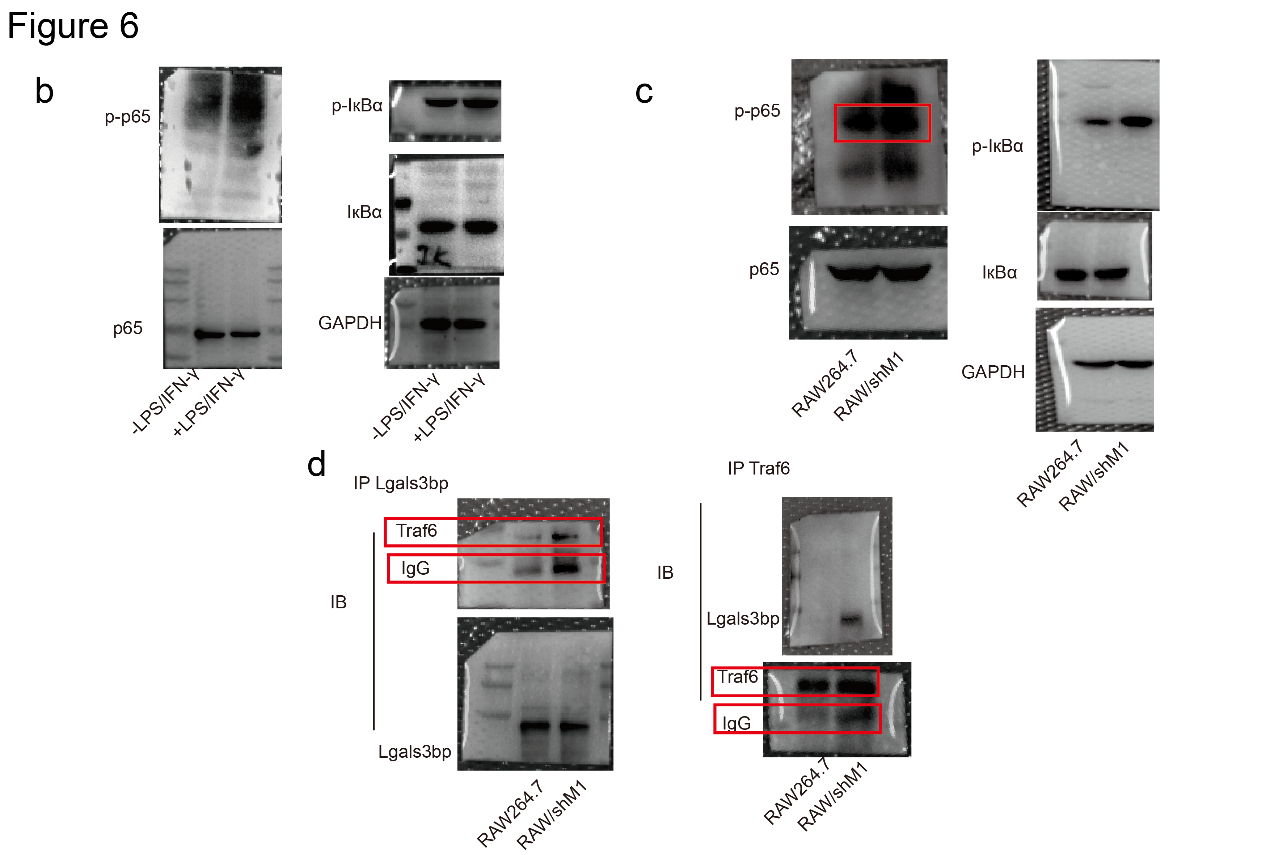


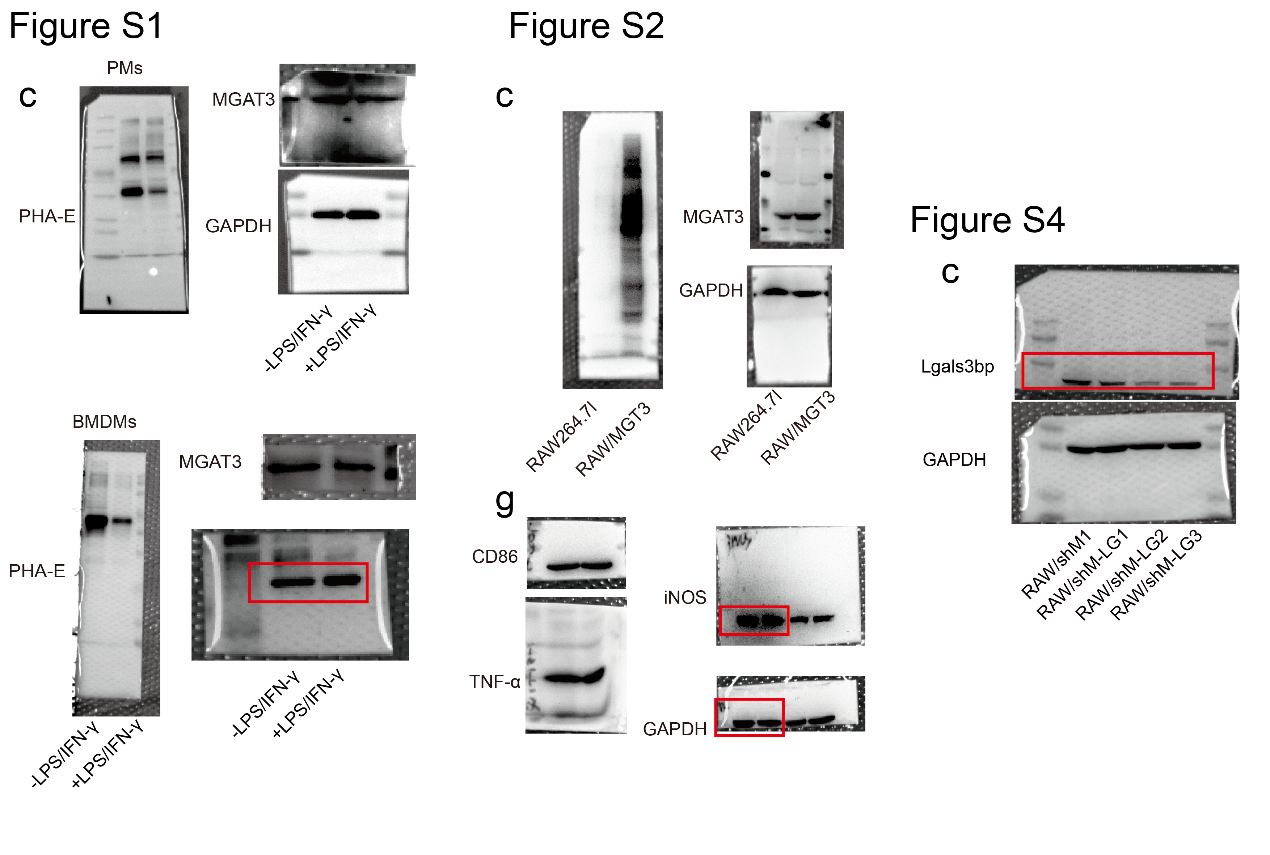


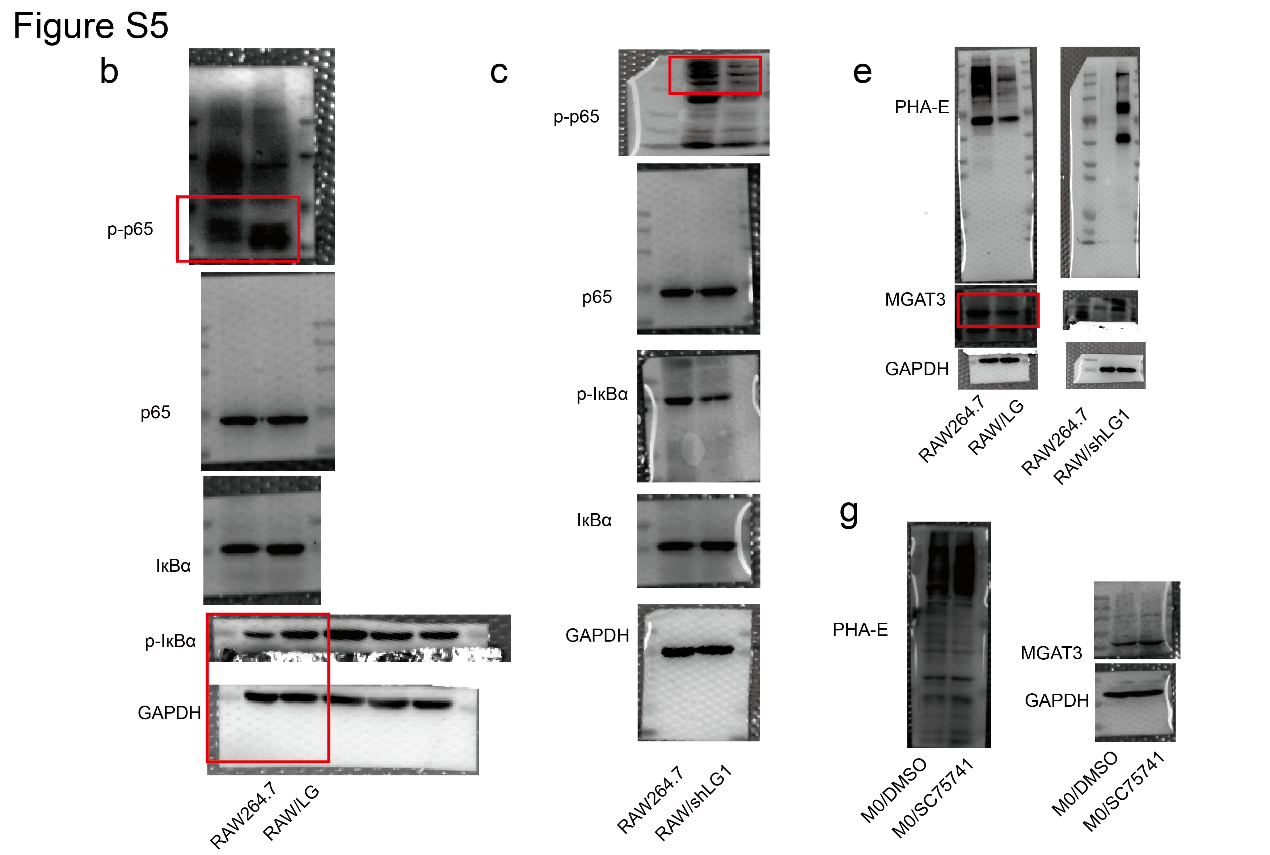

Supplement: Supplementary file 3 — Additional file 2. [file 12964_2023_1432_MOESM2_ESM.docx]
